# Ablation of soft tissue tumours by long needle variable electrode-geometry electrochemotherapy: final report from a single-arm, single-centre phase-2 study

**DOI:** 10.1038/s41598-020-59230-w

**Published:** 2020-02-10

**Authors:** Andrea Simioni, Sara Valpione, Elisa Granziera, Carlo Riccardo Rossi, Francesco Cavallin, Romina Spina, Elisabetta Sieni, Camillo Aliberti, Roberto Stramare, Luca Giovanni Campana

**Affiliations:** 1 University of Padova School of Medicine and Surgery, Padova, Italy; 2 The Christie NHS Foundation Trust, Manchester, UK; 3 Cancer Research UK Manchester Institute, The University of Manchester, Manchester, UK; 4 Veneto Institute of Oncology IOV-IRCCS, Padova, Italy; 5 Department of Surgical Oncological and Gastroenterological Sciences DISCOG, University of Padova, Padova, Italy; 6 Independent statistician, Solagna, Italy; 7 Department of Industrial Engineering, University of Padova, Padova, Italy; 8 Insubria University, Department of Theoretical and Applied Sciences – DiSTA, Varese, Italy; 9 Radiology Unit, Azienda Ospedaliera di Padova, Padova, Italy; 10 Radiology Unit, Department of Medicine DIMED, University of Padova, Padova, Italy

**Keywords:** Cancer, Surgical oncology, Oncology, Cancer, Cancer therapy, Sarcoma, Skin cancer

## Abstract

Standard electrochemotherapy (ECT) is effective in many tumour types but is confined to the treatment of small superficial lesions. Variable electrode-geometry ECT (VEG-ECT) may overcome these limitations by using long freely-placeable electrodes. Patients with bulky or deep-seated soft-tissue malignancies not amenable to resection participated in a single-arm phase-2 study (ISRCTN.11667954) and received a single course of VEG-ECT with intravenous bleomycin (15,000 IU/m^2^) and concomitant electric pulses applied through an adjustable electrode array. The primary outcome was radiologic complete response rate (CRR) per RECIST; secondary endpoints included feasibility, metabolic response, toxicity (CTCAE), local progression-free survival (LPFS) and patient perception (EQ-5D). During 2009–2014, we enrolled 30 patients with trunk/limb sarcomas, melanoma, Merkel-cell carcinoma, and colorectal/lung cancer. Median tumour size was 4.7 cm. Electrode probes were placed under US/TC guidance (28 and 2 patients, respectively). Median procedure duration was 80 minutes. Tumour coverage rate was 97% (29 of 30 patients). Perioperative side-effects were negligible; one patient experienced grade-3 ulceration and infection. One-month ^18^F-FDG-SUV decreased by 86%; CRR was 63% (95% CI 44–79%). Local control was durable in 24 of 30 patients (two-year LPFS, 62%). Patients reported an improvement in “usual activities”, “anxiety/depression”, and “overall health” scores. VEG-ECT demonstrated encouraging antitumour activity in soft-tissue malignancies; a single course of treatment produced high and durable responses, with low complications.

## Introduction

Soft tissue metastases are a frequent event in patients with metastatic melanoma or soft tissue sarcomas (STS) but can also arise in those with breast, lung, and colon cancer^1,2^. Although surgical resection remains the first therapeutic option, locoregional therapies are increasingly being considered by the oncology team with either curative or palliative intent^3–5^. Moreover, the introduction of new active systemic agents has revamped the interest in locoregional therapies and their possible integration into synergistic therapeutic strategies^6,7^.

Over the last decade, electrochemotherapy (ECT) has demonstrated efficacy across a great variety of superficial malignancies, including melanoma, breast, head and neck and gynecologic cancers^8^. In ECT, targeted electric pulses are administered employing small, fixed-geometry electrodes to attain reversible cell membrane electroporation that increases permeability to chemotherapeutics (bleomycin or cisplatin) and local cytotoxic effect^8^. Large multicentre studies indicate ECT as a safe and effective option for the treatment of various cancers, thanks to a multifaceted mechanism of action^9,10^. However, despite its ability to target even widespread superficial tumours, ECT, in its current fashion (i.e. *standard* ECT), does not allow targeting large or deep-seated lesions because of its intrinsic technical limitations related to the size and geometry of electric pulse applicators. According to a meta-analysis including clinical studies on different histologies, the complete response rate (CRR) to ECT reported in tumours larger than 3 cm nearly halved compared to those smaller than 3 cm (33.3% vs 59.5%, respectively)^11^.

Despite these constraints, the principle underpinning ECT - the concurrent administration of chemotherapy and electric pulses to achieve reversible electroporation - stands as a broadly effective approach as demonstrated by the efficacy of *standard* ECT in challenging tumours such as STS and liver malignancies, even though the treatment of these patients required multiple sessions or an open surgical procedure^12,13^. We have previously investigated *standard* ECT in 34 patients with locally advanced or metastatic STS in a phase II study^12^. Notably, despite the large tumour volume (median size, 4 cm) and tissue inhomogeneity, which could have impaired electric current propagation, one-third of patients achieved CR. These favourable results prompted further investigation and the development of new electrode probes, a dedicated pulse generator and, ultimately, a novel ECT modality, i.e. long needle variable electrode-geometry ECT (variable electrode-geometry ECT [VEG-ECT] herein on)^14^. Most notably, VEG-ECT takes advantage of needle electrodes, which are freely placeable and longer than those used in *standard* ECT.

Importantly, these new probes ensure a more extensive and tailored electric field around tumours because they are independent from each other, and their disposition can be adjusted to compose a customised array on the target lesion. This, in turn, may consolidate tumour response, avoid recurrences, and spare patients from retreatment, an event observed in most of the patients treated with *standard* ECT^8^. Miklavcic *et al*. first pioneered the technique in a melanoma patient with a soft tissue metastasis of the thigh, followed by two single-centre studies in patients with liver and bone metastases^12,13,15,16^. Herein, we build on our previous experience with ECT in STS and present novel data assessing the activity, feasibility and safety of VEG-ECT in patients with large or deep soft tissue tumours. Additional endpoints were local tumour control, patient-reported outcomes, and bleomycin pharmacokinetics.

## Patients and Methods

### Patient selection

We prospectively enrolled the patients with measurable locally advanced or metastatic soft tissue tumours of any histotype who were not amenable to resection or unresponsive to conventional treatments according to the local multidisciplinary board. The size of the target lesion had to be comprised between 3 and 7 cm, while maximum tumour depth should not exceed 20 cm, with no infiltration across fascial planes. Eligible patients were adults with an Eastern Cooperative Oncology Group (ECOG) performance status of 0–2 and predicted life expectancy > 3 months, as well as adequate lung and renal function tests. The patients were excluded if any of the following conditions were present: a history of epilepsy, rapidly progressing visceral metastases, active infection, previous bleomycin chemotherapy up to the maximum cumulative dosage (400,000 IU), other local therapies within eight weeks; any systemic treatment four weeks before/after ECT. The protocol was approved by the local Ethics Committee (Comitato Etico Azienda Ospedaliera di Padova, No. 1886P; Supplemental Methods S1), and the study was conducted following the Good Clinical Practice Guidelines and Helsinki declaration (study identifier, ISRCTN.11667954). Treatment indication was agreed within the dedicated multidisciplinary team, and all patients provided signed informed consent.

### Preoperative assessment and treatment planning

**Figure 1:**
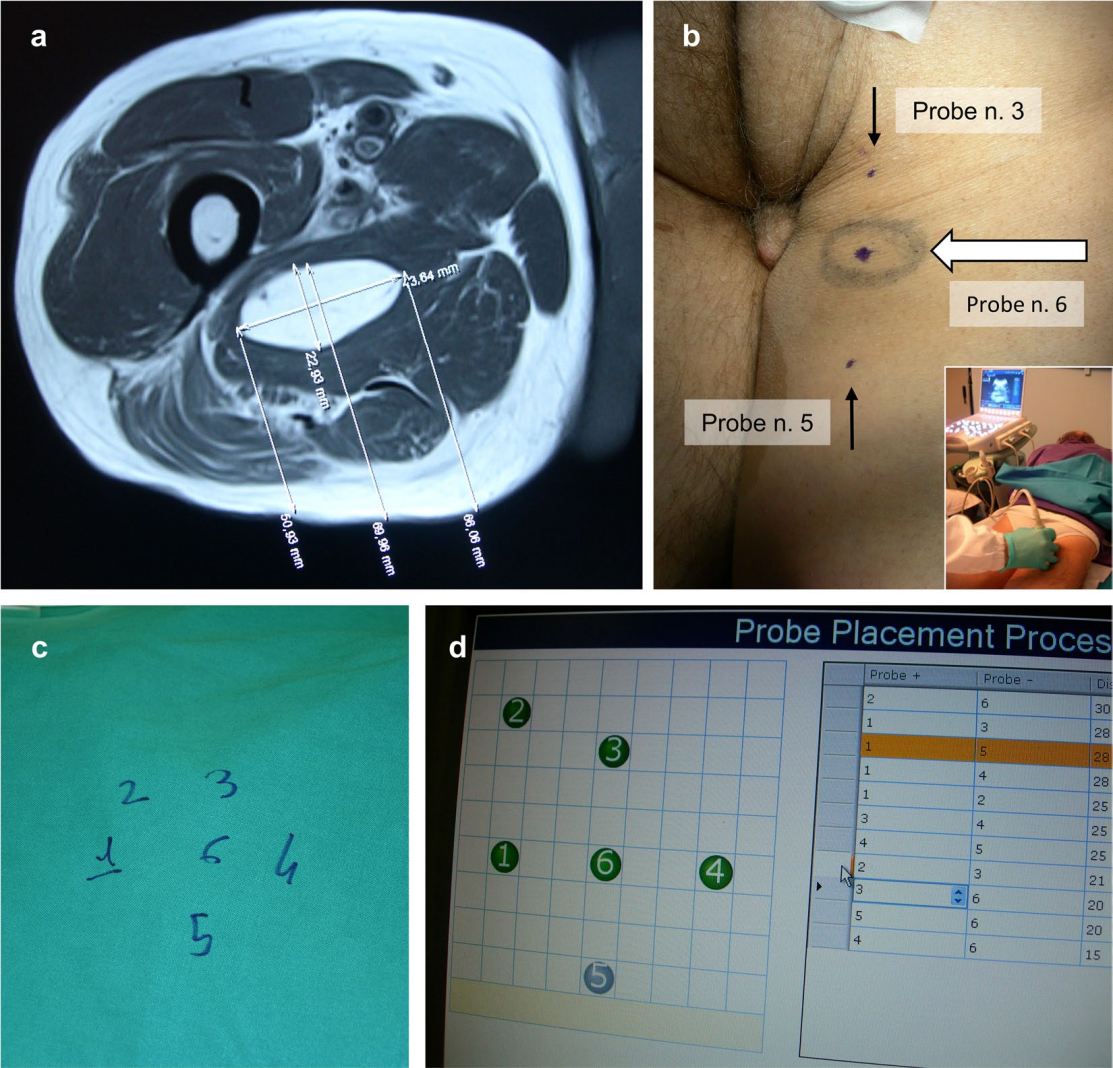
Treatment planning in a patient with malignant peripheral nerve sheath tumour (MPNST) of the posterior compartment of the thigh and stable lung metastases following systemic treatment and stereotactic radiotherapy. (**a**) Baseline MR scan demonstrating a 4-cm ellipsoidal intramuscular mass and a practicable approach for electrode insertion. (**b**) Preoperative US study (insert) and placement of skin marks at the site of electrode insertion. (**c**) Intraoperative sketch of the electrode array composition, including one central and five peripheral probes. (**d**) Following electrode insertion, the final position and actual distance of each electrode pair are uploaded into the software of the pulse generator to customise voltages.

Patients underwent baseline evaluation through CT/MR and ^18^F-PET-CT scan. ECT treatment plan relied on preoperative radiologic imaging, which included the following parameters: the most feasible approach for electrode insertion, the number of electrode probes (minimum two, maximum six, according to the characteristics of the pulse generator), their geometric configuration, and the distance between probes of each electrode pair. We aimed at reducing the total number of probes while ensuring optimal tumour coverage. When dealing with tumours indicatively larger than 27 cm^3^, the electrodes were retracted and reactivated along the same track to encompass the whole target volume, and additional placements were planned to extend the ablation volume further when required. Whenever possible, a 0.5–1 cm treatment safety margin was included to eradicate microscopic infiltration. The target lesion was assessed preoperatively by a radiologist to confirm the reliability of ultrasound (US) scan as a means to guide electrode placement in the operating theatre (Fig. 1); conversely, when US-based electrode tracking was deemed unsatisfactory, the procedure was scheduled in the interventional radiology suite, and the insertion of probes was performed under TC guidance.

### Procedure

**Figure 2:**
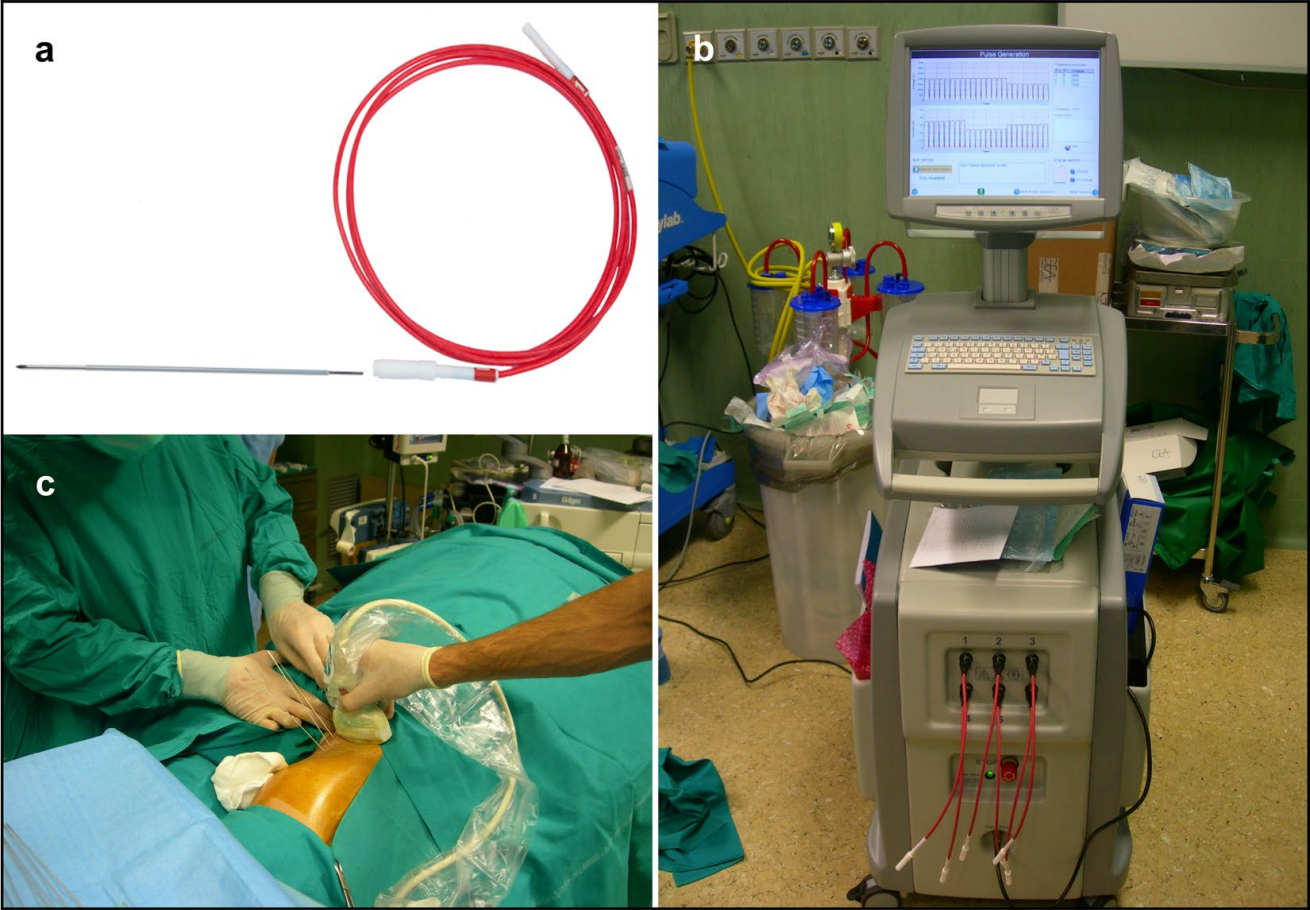
VEG-ECT equipment and intraoperative electrode insertion. (**a**) An electrode probe. The needle electrode is 1.2-mm thick and shielded by an insulating layer except on the distal extremity (active tip), which is available in different lengths, ranging from 20 to 50 mm. (**b**) The pulse generator. The device can manage a maximum of six probes simultaneously to compose the electrode array. (**c**) Percutaneous electrode placement under US guidance in a patient with soft tissue sarcoma of the lower limb (Fig. 1). The maximum distance between electrodes cannot exceed 3 cm due to safety reasons and the needle probes must be placed in a parallel fashion to ensure homogeneity to the electric field.

**Figure 3:**
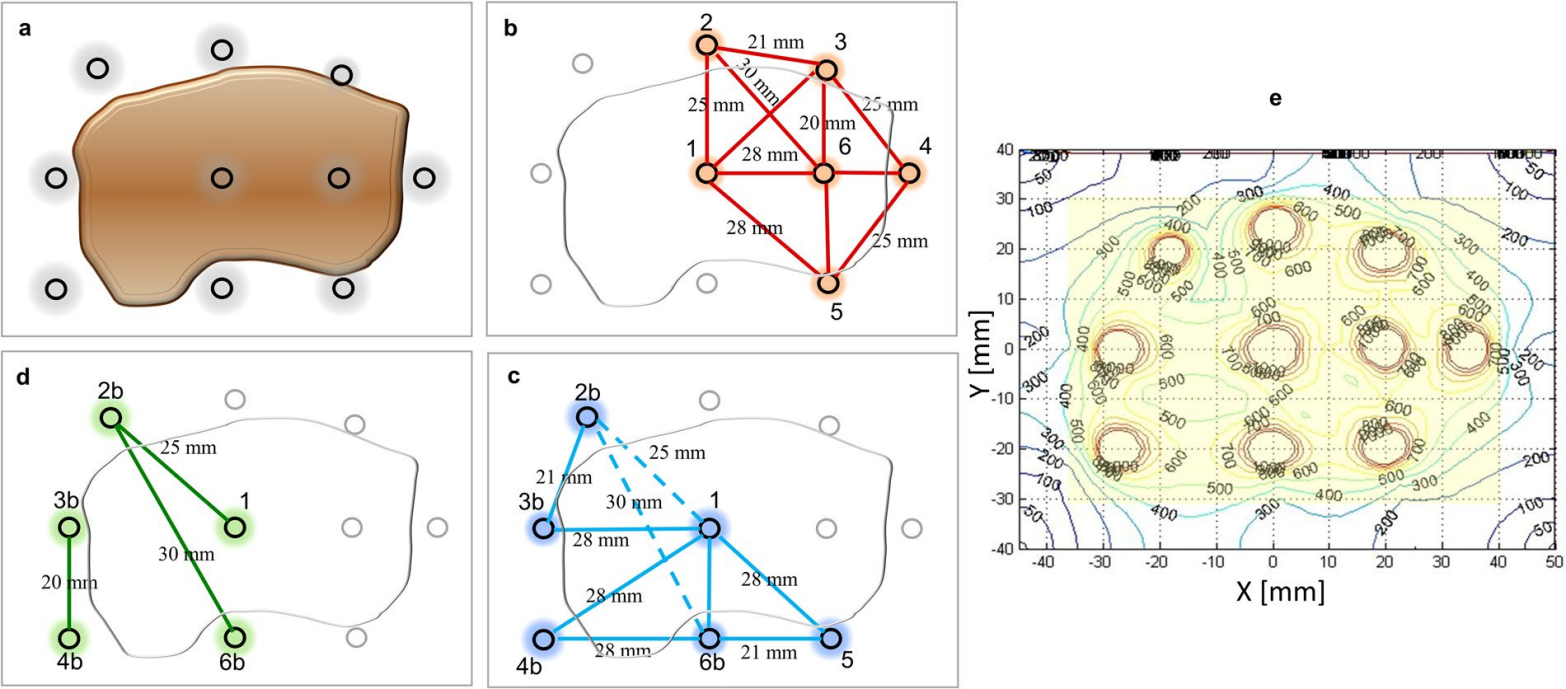
Sequential electrode activation and post-treatment check. (**a**) Pretreatment plan of a 6 × 3 × 3 cm tumour using two placements of the whole electrode array and a total of ten electrode probes (each probe has a 4-cm active tip). (**b–d**) Placement of the electrode array and activation of electrode pairs. The colour lines indicate current flow, while dotted lines indicate suboptimal electric current (lower than 1.5 A). After each pulse delivery, the electrodes were partially retracted and reactivated to complete coverage of the target volume. (**d**) Three electrode pairs are re-activated to offset the suboptimal electric current during the previous pulse delivery. (**e**) Treatment verification. Distribution of the electric field intensity based on treatment-specific electrical parameters recorded by the software of the pulse generator (threshold for reversible tumour electroporation, 400 v/cm).

The procedure was carried out under tailored anesthesiological regimens, depending on tumour features and patient preference. The administration of bleomycin was in keeping with the European Standard Operating Procedure of ECT (ESOPE), with a slow intravenous infusion of 15,000 IU/m^2^ (8). After eight minutes, the electric pulses were delivered using 20 cm-long needle electrodes with a 1.2-mm diameter, connected to a CE certified pulse generator (Cliniporator VITAE, IGEA, Carpi, Italy). Each electrode was inserted into or around the target lesion under US or TC guidance with the support of an on-site radiologist (Fig. 2). Based on previously published experience, we initially approached tumours with an array of five or six electrode probes to obtain optimal tumour coverage and avoid recurrences (15). The treatment was preceded by a train of low-voltage pulses (test phase) to verify the actual flow of the electric current between each electrode pair and the failing probes were repositioned until achieving an adequate current flow. During treatment, the voltage amplitude between each couple of electrodes could range from 500 V to 3,000 V depending on their distance. In each application, electric pulses were delivered over some tens of seconds. When required, the electrode array was juxtaposed multiple times according to pre-treatment plan to achieve tumour coverage (Fig. 3). Throughout the study and every three procedures, procedural details and patient outcomes were reviewed in an audit meeting.

### Outcome evaluation

The feasibility of the procedure was graded as treatment delivery success rate (i.e. the number of patients in whom the target tumour was covered with electric fields exceeding the threshold for reversible electroporation). This assessment implied both an intra-operative evaluation of the electric current produced between electrode pairs (Supplementary Fig. S1) and post-operative evaluation of the distribution of the electric field intensity around the tumour (Supplementary Fig. S2). Tumour response assessment comprised a metabolic evaluation with ^18^F-PET-CT performed between postoperative day 28–35, and radiologic evaluation using CT/MR (performed at 30 and 60 days following the Response Evaluation Criteria in Solid Tumors [RECIST] v1.1). Subsequent radiologic evaluations were agreed with the referring medical oncologist every three/four/six months depending on disease course, patient status and eventual ongoing treatments. Patients were followed up at one week, 1, 2, 3 and 6 months, and every three months thereafter; those with ulcerated tumours were followed up by a nursing team for wound dressing. Toxicity was scored according to the Common Toxicity Criteria for Adverse Events (CTCAE v4.03). Patient-reported outcomes were collected at baseline, one month and two months utilising the EuroQoL quality of life scale (EQ-5D-3L) of the Euro Quality of Life Group (https://euroqol.org/eq-5d-instruments/eq-5d-3l-about/).

### Bleomycin pharmacokinetics

Blood and tumour samples were collected to evaluate bleomycin concentration through high-performance liquid chromatography (HPLC). Two 10 ml venous blood samples were taken at 8 minutes and 28 minutes after bleomycin infusion and centrifuged at 3000 rpm for 10 minutes; collected plasma was stored frozen at −20 °C. A tru-cut biopsy of the tumour was performed 8 minutes after chemotherapy infusion. The specimens will be analysed using a recently introduced and more reliable technique developed by another research group^17^, and results will be presented separately.

### Study design

This single-arm phase 2 study was conducted using an exact single-stage design, according to Fleming^18^. The primary endpoint was antitumor activity in terms of CR rate. With a type I error of 0.05 and a power of 0.80, we set the maximum accrual at 30 patients, the largest response probability at 0.65 and the smallest response probability at 0.40. The acceptance threshold for efficacy was CR achievement in 17 or more patients.

### Statistical analysis

Continuous data were compared using the Mann-Whitney test or the Kruskal-Wallis test; Δ-SUV values were evaluated with the Wilcoxon test and the correlations with the Spearman’s rank correlation coefficient. Categorical data were compared using Fisher’s exact test. ECOG and EQ-5D-3L scores were evaluated by one-way repeated measures ANOVA with rank transformation, and variation in pain levels with pairwise Fisher’s test with Bonferroni adjustment for multiple comparisons. Local progression-free survival (LPFS) was defined as the time from treatment administration to in-field disease progression. Survival curves were estimated with the Kaplan-Meier method. The effects of the duration of the procedure and CR achievement on LPFS were investigated with Cox regression models. The proportional hazard hypothesis was verified using Schoenfeld residuals (with P = 0.68 for local response and P = 0.52 for procedure duration, where P > 0.05 confirms the assumption of proportionality). A P-value lower than 0.05 was considered statistically significant. Analyses were performed using R 3.5 (R Core Team 2018. R: A language and environment for statistical computing. R Foundation for Statistical Computing, Vienna, Austria. Available online at https://www.R-project.org/).

## Results

### Series

We enrolled 30 patients between September 2009 and October 2014. Most of them had malignant melanoma or STS (43% each) (Table 1). Fifteen patients (50%) had visceral metastases, nine (30%) had also skin tumour involvement. Median tumour size was 4.7 cm (range, 3.0–7.0), with lower values in tumours located in the lower limbs (*P* = 0.002). Three patient were receiving systemic treatment at the time of the VEG-ECT: these included a melanoma patient under targeted therapy and two sarcoma patients who were receiving paclitaxel with a mixed response (i.e. confirmed stable disease [SD] or partial response [PR] on visceral metastases but symptomatic progression on soft tissue as per RECIST criteria).

**Table 1:** Cohort clinical characteristics (n = 30).

Characteristics	Median (range)or No. (%)
**Age** (yrs.)	67 (28–88)
**Sex**	
Female / male	18 (60)/12 (40)
**BMI** (Kg/m^2^)	24.5 (18.1–35.1)
**Tumour histotype**	
Soft tissue sarcoma^*^	13 (43)
Melanoma	13 (43)
Merkel cell carcinoma	2 (8)
Lung adenocarcinoma	1 (3)
Colon adenocarcinoma	1 (3)
**Disease presentation**°	
Primary^†^	3 (10)
Recurrent/metastatic	27 (90)
**Synchronous metastases**°	
Skin	9 (30)
Lymph node	5 (17)
Visceral^‡^	15 (50)
**Previous treatments**	
Surgery	26 (87)
Radiotherapy	10 (33)
Systemic treatment	21 (70)
**Interval to diagnosis** (months)	26.3 (0–137)
**Target tumour location**	
Trunk	6 (20)
Upper limb	5 (17)
Lower limb	19 (63)
**Tumour burden**	
No. of target lesions per patient	1 (1–3)
Pts with single/multiple lesions	25 (83) / 5 (17)
Size (cm)	4.5 (3.0–7.0)
upper limb	3.5 (3.0–6.0)
lower limb	4.5 (3.5–6.0)
trunk	6 (4.0–7.0)
**Target tumour depth (cm)**	
Minimum^⁋^	2.0 (0.5–4.5)
Maximum^§^	3.3 (1.0–10.5)
Subcutaneous/subfascial	23 (77) / 7 (23)
**Other features**	
Previous treatments^#^	20 (70)
Ulceration	11 (37)
Bleeding	7 (23)
SUV	9.6 (5.40–16.57)

Legend: BMI, body mass index; ECT, electrochemotherapy; SUV, standard uptake value at 18F-Positron Emission Tomography (PET).

^*^Sarcoma histotypes included the following: malignant peripheral nerve sheath tumor (n = 3), angiosarcoma (n = 1), clear cell sarcoma (n = 1), epithelioid sarcoma (n = 1), fibrosarcoma (n = 1), leiomyosarcoma (n = 1), liposarcoma (n = 1), myoepithelioma (n = 1), rhabdomyosarcoma (n = 1), synovial sarcoma (n = 1), undifferentiated pleomorphic sarcoma (n = 1).

°At enrolment.

^†^Two soft tissue sarcoma patients had synchronous distant metastases and underwent ECT on their primary tumour as a form of palliative therapy; one patient with locally advanced primary soft tissue sarcoma received ECT with neoadjuvant intent.

^‡^Location of the metastatic disease included the following: lungs (n = 11 patients), liver (n = 3) and adrenal gland (n = 1).

^⁋^Distance between the skin surface and the upper margin of the tumour (calculated on 15 deep-seated tumours with no skin infiltration or fungating spread).

^§^Distance between the skin surface and the deepest tumour margin.

^#^Previous treatments on the target tumor included the following: cytotoxic chemotherapy (n = 10 patients), radiotherapy (n = 5), surgery (n = 3), limb perfusion (n = 2), targeted therapy (n = 1).

### Treatment

The procedure was completed in all cases. Treatment parameters are summarised in Table 2. The intent was palliative in all but one patient, who received neoadjuvant VEG-ECT, followed by curative surgery. The median duration of the procedure was 80 min (range, 40–130), with no significant difference between subcutaneous and subfascial tumours (80 min [range, 45–120] vs 70 min [range, 50–130], *P* = 0.95). We used a median of two placements of the electrode array (range, 2–4), and the number of placements correlated with the size of the treated tumour (Rho 0.54, *P* = 0.002). In 18 patients (60%), one or more electrode probes had to be repositioned due to suboptimal electric current (Supplementary Fig. S1). The post-procedural review of treatment data stored in the software of the pulse generator indicated that tumour coverage with electric fields was not achieved in one of 30 patients (Supplementary Fig. S2), thus leading to a treatment delivery success rate of 97%. Four patients with malignant melanoma (n = 3) or STS (n = 1) received concurrent *standard* ECT within the same procedure for synchronous skin metastases. Following the on-trial audit meetings, we implemented some technical and procedural details. First, we introduced an echogenic trocar tip to electrode design to facilitate its insertion and enable US tracking within tissues. Second, we introduced some dedicated devices to stabilise the electrode array and to avoid the displacement of electrode probes due to muscle contraction (Supplementary Fig. S3). Finally, to prevent any delay resulting from electrode repositioning and maximise tumour exposure to chemotherapy within the therapeutic window of bleomycin (20–40 minutes), we postponed chemotherapy infusion at the end of the “test pulse” phase.

**Table 2:** Treatment (n = 30 procedures).

Parameter	Median (range) or No. (%)
**Setting**	
Operating theatre	28 (93)
Interventional radiology suite	2 (7)
**Duration of the procedure** (min)^*^	80 (40–130)
**Type of anaesthesia**	
General	12 (40)
Sedation	3 (10)
Spinal	15 (50)
**Bleomycin dose** (U)°	27 (24–30)
**Electrode array composition**^†^	
Five electrodes	13 (43)
Six electrodes	17 (57)
**Electrode array placements**^**‡**^	2 (1–4)
**Probe repositioning**^**⁋**^	18 (60)
**Distance between electrodes** (mm)	21 (10–30)
**Applied voltage** (V)	1800 (700–3000)
**Pulse duration** (μs)	100
**Pulse repetition frequency (Hz)**	1,000
**Electric current** (A)	26.4 (18.3–41.0)
**Resistance (Ω)**	72.8 (50.2–144.9)

*The duration of the procedure was retrieved from electronic medical records and calculated as the interval between the end of the preparatory phase of anaesthesia (whatever the technique applied) and the end of the application of electric pulses.

°The bolus of bleomycin (15,000 IU/m^2^) was administered intravenously over one minute.

^†^The number of electrodes correlated with tumour size (P = 0.002); in the five-electrode group, the median tumour size was 45 mm (range, 35–55), while in the six-electrode group it was 55 mm (range, 40–70 mm).

^‡^Number of planned applications of the whole electrode array to treat the target tumour.

^⁋^Patients who needed unplanned reinsertion of one or more probes during the procedure due to suboptimal current flow in the test pulse phase or during treatment delivery.

### Safety and toxicity

There were no serious adverse events, either intraoperatively nor during the hospital stay, which lasted a median of 2 days (range, 1–4) (Supplementary Table S1, and Figs. S4, S5). A melanoma patient with a subcutaneous tumour in the lumbar region experienced intraoperative grade-2 bradycardia and received treatment with atropine; her following progress was uneventful.

### Post-treatment pain

Overall, post-treatment pain was fluctuating (Supplementary Fig. S5). In particular, after ECT, nine patients (30%) experienced no pain, whereas 16 (53%) and 5 (17%) reported mild and moderate pain, respectively. Pain level declined during hospital stay (*P* = 0.02), increased after one month (*P* = 0.002), and finally improved at two months (*P* = 0.02). At six months, only 8/29 evaluable patients had mild/moderate pain compared to 16/30 at baseline (*P* = 0.04). One melanoma patient, who underwent treatment for a proximal arm metastasis, reported grade-2 sensory neuropathy, which lasted three months and required corticosteroids, gabapentin and vitamin B12. Higher pain scores in the CTCAE scale and the EQ-5D questionnaire were associated with the increasing number of electrode placements (*P* < 0.001), previous chemotherapy exposure (*P* = 0.005), and use of six vs five probes (*P* = 0.04); we observed no association between postoperative pain and the anesthesiological technique (Supplementary Table S2).

### Local toxicity

Throughout hospital stay, 16 patients (54%) reported mild adverse events (Supplementary Table S1). Subsequently, there were no relevant toxicities, except for grade-3 skin ulceration and soft-tissue infection in a patient with an STS of the buttock (Supplementary Fig. S4). Higher local toxicity was observed in patients with sarcoma (at 1- and 2-month follow-up, *P* = 0.03 and *P* = 0.03, respectively), baseline tumor ulceration (*P* = 0.002; *P* = 0.001), longer duration of the procedure (*P* = 0.007; *P* = 0.04), application of six vs five electrodes (*P* = 0.03), and multiple electrode placements (*P* = 0.04). CR achievement was not associated with higher toxicity (Supplementary Table S3). Before ECT, the target tumours were irradiated more frequently in patients with sarcoma than in those with melanoma (9/13 vs 0/13, P < 0.001); additionally, there were no significant differences between these subgroups regarding the following variables: previous systemic treatment (P = 0.20), tumour size (P = 0.79), tumour depth (P = 0.98), baseline tumour ulceration (melanoma, 2/13; sarcoma, 6/13, P = 0.20), and response to ECT (P = 0.69).

### Tumour response

#### Metabolic evaluation

**Figure 4:**
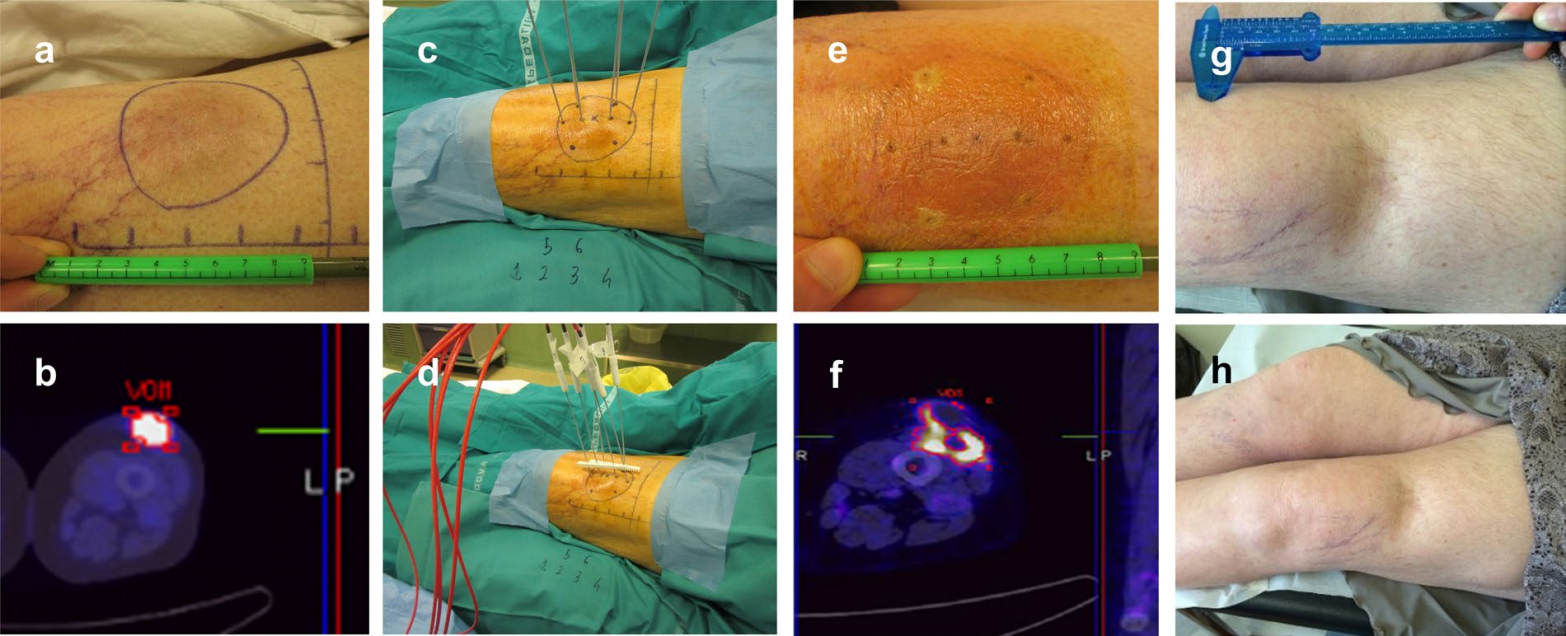
Tumour response in a melanoma patient. This woman presented with a 4-cm soft tissue metastasis in the anterior aspect of the thigh following previous systemic treatment and bilateral adrenalectomy for metastatic disease. (**a**) Baseline clinical presentation and (**b**) PET-CT scan. (**c**) Intraoperative electrode insertion (the treatment plan included two separate electrode placements to encompass the target volume). (**d**) Connection of the electrode probes to the pulse generator. (**e**) Early post-treatment erythema. (**f**) One-month PET-CT scan showing marked metabolic response and residual inflammatory features at the border of the ablation zone. (**g**) Six-month and (**h**) 2-year clinical follow-up.

The median SUV decreased from 9.6 (IQR 7.9–12.6) at baseline to 1.6 (IQR 0.0–2.4) at 1 month (*P* < 0.001), with a median Δ-SUV of 86% (IQR, 74–100%) (Fig. 4). One patient who achieved a near-complete metabolic response (Δ-SUV, 90%) underwent curative resection after eight weeks, and the surgical specimen revealed pathological evidence of more than 80% of tumour necrosis. CR rate was 33% (10 of 30 patients). Among the 20 patients with residual FDG uptake at 1-month PET-CT, 9 (45%) finally achieved CR at 2-month radiological evaluation. In these nine patients, the median Δ-SUV was 86% (range, 73–90%). Two of the nine patients with residual metabolic uptake at one month (Δ-SUV 90.9 and 77.8, respectively) and complete radiological response at two months developed local recurrence in the ECT field during follow-up.

#### Radiologic evaluation

At one-month evaluation, CR was observed in 19 patients (63%, 95% CI 44–79%), PR in 8 patients (27%), and SD in 3 patients (10%). At two months, CR was confirmed in all 19 patients (63%, 95% CI 44–79%), PR was achieved in nine patients (30%), and SD in two patients (7%). Higher CR rates were reported in small-size (*P* = 0.005) and limbs tumors (*P* = 0.01, Table 3). Eighty-five per cent of patients treated using five electrodes achieved CR compared to 47% of those treated with six electrodes (*P* = 0.06). Finally, 75% of chemotherapy-naïve patients achieved CR, compared to 50% of those previously exposed to chemotherapy (*P* = 0.40). CR rate in melanoma and STS were similar (9/13 [69.2%] vs 7/13 [53.8%], *P* = 0.69).

**Table 3:** Tumour response according to patient characteristics and treatment parameters.

Characteristic	Response		*P*
	SD/PR* (n = 11)	CR* (n = 19)	
**Histotype**			0.69
Melanoma	4 (31)	9 (69)	
Sarcoma	6 (46)	7 (54)	
**Anatomical location**			0.01
Upper limb	0	5 (100)	
Trunk	5 (83)	1 (17)	
Lower limb	6 (32)	13 (68)	
**Tumour size (mm)**	55 (50–60)	45 (40–48)	0.005
**Tumour ulceration**			0.99
No	7 (37)	12 (63)	
Yes	4 (36)	7 (64)	
**Tumour depth**			
Subcutaneous	10 (57)	13 (43)	0.11
Subfascial	7 (14)	6 (86)	
**Previous Tx on target tumour**			0.99
No	3 (33)	6 (67)	
Yes	8 (38)	13 (62)	
None or surgery	3 (25)	9 (75)	0.40
CT/ILP	6 (50)	6 (50)	
**Visceral metastases**			0.99
No	6 (40)	9 (60)	
Yes	5 (33)	10 (67)	
**No. of electrode probes**			0.06
Five	2 (15)	11 (85)	
Six	9 (53)	8 (47)	
**No. of electrode placements°**			0.27
1 ≥ 2	0	4 (100)	
	11 (42)	15 (58)	
**Electrode repositioning**^**†**^			0.44
No	3 (25)	9 (75)	
Yes	8 (44)	10 (56)	
**ECT duration (min)**	80 (73–110)	75 (60–93)	0.14

SD, stable disease; PR, partial response; CR, complete response; Tx, treatment; CT, chemotherapy; ILP, isolated limb perfusion.

*Data expressed as number (%) or median (IQR).

°Number of planned placements of the whole electrode array to cover the target volume.

^†^Patients in whom it was necessary to replace one or more probes due to low electric current.

#### Local tumour control

Median follow-up time was 17.6 months (range, 13.1–93.4). Twenty-three patients (77%) received further oncological treatments for the occurrence of local, locoregional or systemic progression. These treatments were administered after a median of 7 months (range, 4–36) and included chemotherapy (n = 17 patients), immunotherapy (n = 4), local palliative radiotherapy (n = 3), targeted therapy (n = 2), surgery (n = 1), standard ECT (n = 1), and isolated limb perfusion (n = 1). The local failure rate was 27% (8/30), after a median of 8.1 months (range, 3.1–20.7). Of these 25 patients, 8 experienced a recurrence in the ECT field, and 3 of these 8 patients received systemic post-ECT treatment before the recurrence.

**Figure 5:**
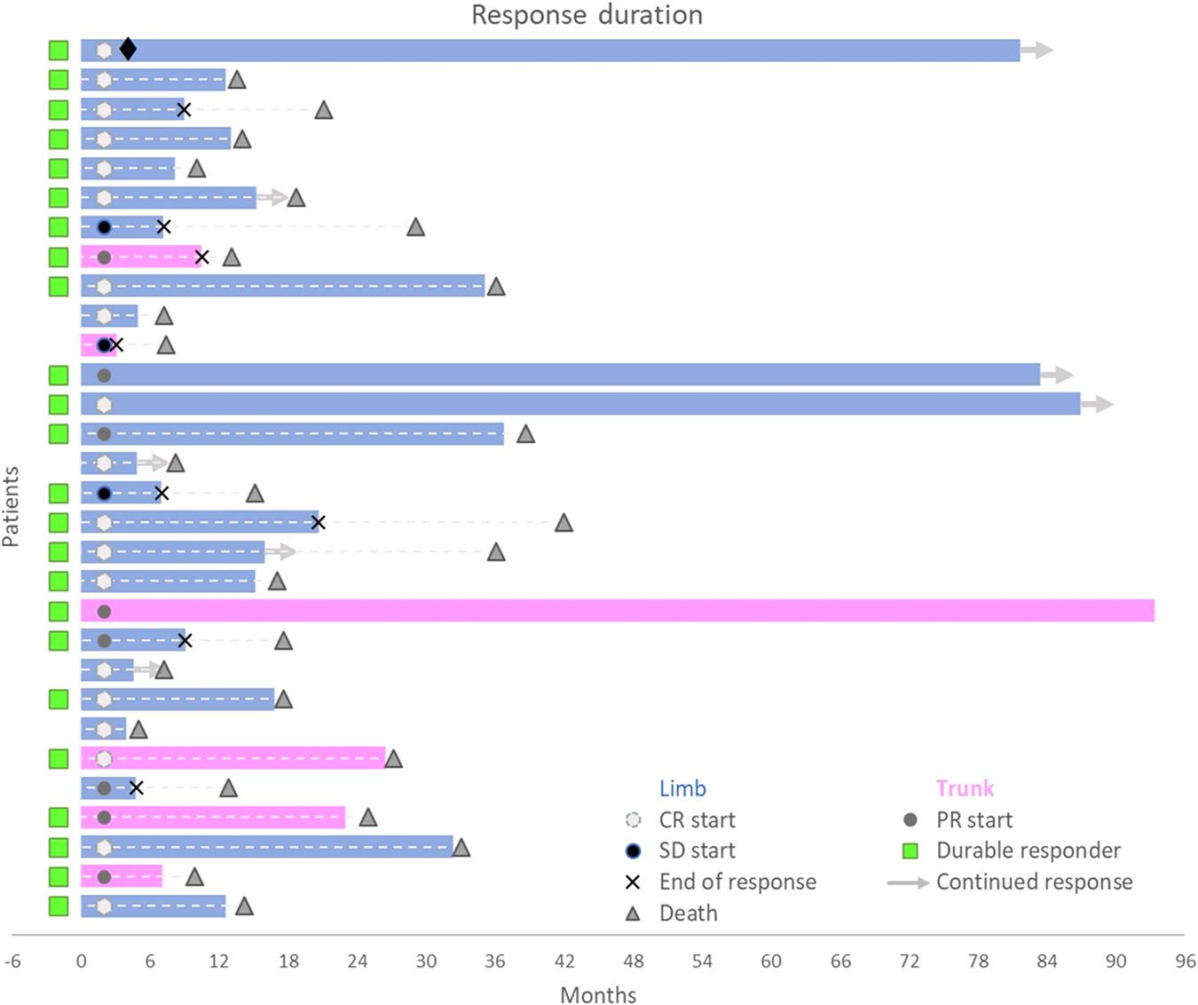
Swimmer plot graph of response duration. Each bar represents a patient, and its length is proportional to the duration of local tumour control. Twenty-three of 30 patients received further oncologic treatments following VG-ECT after a median of 7 months (range, 4–36). These included the following: cytotoxic chemotherapy, n = 17; immunotherapy, n = 4; radiotherapy, n = 3; targeted therapy, n = 2; standard ECT, n = 1 surgery, n = 1; ILP, n = 1). The patient represented by the first upper bar underwent surgical resection (♦) of the target lesion treated with VG-ECT. *Legend*: CR, complete response; durable responder, a patient with CR/PR/SD persisting for at least six months; PR, partial response; ILP. Isolated limb perfusion; SD, stable disease.

A durable response (≥6 months) was achieved in 24 out of 30 (80%) patients (Fig. 5). One- and 2-year LPFS was 72% and 65%, respectively (Supplementary Fig. S6). CR achievement was associated with prolonged LPFS (HR 0.17, 95% CI 0.03–0.85; *P* = 0.01), while a longer duration of the procedure seemed to be inversely associated with LPFS (HR 1.03, 95% CI 1.00–1.06; *P* = 0.05).

#### Performance status and patient-reported outcomes

Over the first two months, we observed a significant decrease in the ECOG score (Supplementary Fig. S7) as well as in the “usual activities”, “anxiety/depression” and VAS scores of the EQ-5D-3L questionnaire (Supplementary Fig. S8).

#### Patient outcome

Seventeen patients (57%) developed new metastases in the skin/soft-tissue, after a median interval of 6 months (IQR 5–9). Twenty-six (87%) patients experienced systemic progression after a median of 8 months (IQR 5–11) and died after 15 months (IQR 11–27) (Fig. 5). One patient developed fatal acute respiratory distress syndrome five months after treatment. Some patients had remarkably prolonged survival, with eight subjects (27%) still alive more than three years after ECT (Supplementary Fig. S6).

## Discussion

The results of this first-in-field study demonstrate that VEG-ECT can overcome two well-known limitations of *standard* ECT - large tumour size and deep anatomical location - and may represent an active and safe locoregional therapy for patients with soft tissue tumours when other treatments are not practicable. Over the last few years, electroporation-based procedures have evolved considerably^8,14^. Among the currently available approaches (*standard* ECT, irreversible electroporation [IRE], calcium electroporation, VEG-ECT and gene electrotransfer [GET]), *standard* ECT has become the most popular technique in clinical practice thanks to the ease of application, efficacy and safety coupled with an increasing number of indications^8,14^. However, the standard equipment does not allow targeting bulky (i.e., >3 cm) and deep-seated (i.e., >3 cm from the skin plane) tumours.

Our findings provide new insights into a novel electroporation-based treatment, VEG-ECT, and support the results of two previous studies in patients with liver and bone metastases. Edhemovic *et al*. reported an 85% CR rate with no relevant side effects in 16 patients with colorectal liver metastases (median tumour size, 1.7 cm) who were treated by either VEG- (n = 13) or *standard* (n = 3) ECT during an open surgical procedure^13^. In 2016, Bianchi *et al*. reported on 29 subjects with painful bone metastases who underwent one or repeated (34% of patients) VEG-ECT sessions under fluoroscopy or CT guidance^16^. The authors observed disease stabilisation in most cases and a significant improvement of pain control in 84% (20/24) of patients.

In the present study, the response rate after a single course of treatment was 93%, which is in line with our previous research on *standard* ECT in superficial STS, in which we treated 34 patients with a median tumour size of 4 cm and achieved a 92% response rate (12). Most notably, the results from the present study indicate a nearly doubled CRR (63% vs 32%), which was obtained through a single treatment session - whereas half of *standard* ECT patients underwent retreatment - and low complications. Importantly, the high rate of resolved tumours, which is likely the effect of the improved tumour coverage with electrodes, may reduce the need for retreatments and the associated toxicity, as demonstrated by the lower occurrence of side effects compared to *standard* ECT, where one-third of patients faced grade-3 toxicity^12^.

Although the functional disability caused by soft tissue metastases is generally mild, still they can impair patient quality of life due to day-to-day experience, progressive growth and ulceration as observed in more than one-third of our patients (Table 1). Since these lesions are a hallmark of advanced disease^1^, any palliative intervention should cause as little morbidity as possible, and the indication to treatment should be judicious. Interestingly, patient-reported outcomes in our study confirm the tolerability of VEG-ECT and suggest a potential palliative benefit.

Moreover, due to the novelty of the technique, incremental improvement and amelioration of its therapeutic ratio can be expected. According to our data, the number of probes and the number of placements of the whole electrode array correlated with post-treatment pain and local toxicity (Supplementary Table S2, S3). This observation suggests that researchers should exploit technical advancements aimed at reducing the invasiveness of the procedure (e.g. by reducing the number of probes through image-guided treatment planning or by using deployable electrode probes) to pursue future clinical application and maximise patient benefit^14^.

Remarkably, we achieved a durable remission in 80% of our cases (Fig. 5), which is a positive result in such an advanced disease setting, although the contribution of subsequent oncologic treatments and the follow-up schedule could have influenced the patient outcome. Local tumour control was slightly lower compared to our previous experience with *standard* ECT (2-year LPFS, 62% vs 72.5%); however the two populations are not comparable; moreover, we believe that, in selected cases, retreatment could have been proposed safely - although out of the study protocol - to prolong tumour control. However, the feasibility and safety of retreatment with VEG-ECT needs confirmation^16^.

At present, the clinical experience with this technique is still limited. In their pioneering study, Miklavcic *et al*. treated a 2-cm soft tissue metastasis from melanoma in a lower limb using four probes and achieved a 50% pathological response^15^. By comparing pre-treatment planning with measurements taken during the procedure, they revealed that small inaccuracies in electrode placement were responsible for suboptimal (i.e. 94%) tumour coverage. This observation has important implications for clinical practice because large tumour size is a common feature of soft tissue malignancies and a critical aspect in the application of electroporation-based treatments. An analysis of patients with soft tissue metastases treated at the Ohio University Medical Center revealed that 32% (38/118) had lesions larger than 3 cm, while in the experience of the Royal Orthopaedic Hospital of Birmingham, 78% (78/100) of patients had tumours exceeding five centimetres^2,19^. Finally, we confirm here that tumour size remains significantly associated with response to ECT, also when using the VEG modality (Table 3).

As expected from previous studies with ECT, the only toxic effects were local site reactions. Of note, however, bulky lesions may develop a considerable amount of tissue necrosis due to ECT sustained antitumour activity, with the inherent risk of ulceration, infection, and delayed healing (Supplementary Table S1). Although numbers are too small to draw firm conclusions, we found that local toxicity correlated with pre-existing tumour ulceration, sarcoma histotype, number of electrode placements as well as the duration of the procedure (Supplementary Table S3). The higher toxicity recorded in sarcoma patients could be explained by the more frequent use of radiotherapy in this subgroup, coupled with the higher presence of baseline tumour ulceration. Overall, these observations could inform patient selection in future studies and help to improve the palliative benefit of VEG-ECT by excluding or informing the subjects with ulcerated or skin-infiltrating tumours, reducing bleomycin doses in those previously irradiated, or implementing treatment planning through the reduction of the number of electrode probes.

Nowadays, thanks to the continuous improvement of patient survival produced by systemic therapies, soft-tissue metastases are being encountered more frequently. They occur in 1.2–6.6% of STS patients^1,19^ and, together with skin and lymph nodes, represent a primary site of diffusion of cutaneous melanoma^20–23^, and a possible modality of spread for carcinomas (e.g. lung, breast, kidney, and colon) and lymphoproliferative disorders^1,2,19,24^. The management of these lesions is challenging because there are few clear-cut solutions and a multitude of therapeutic options. Generally, surgical resection deserves primary consideration, particularly in patients with oligometastatic disease, but systemic treatment still represents the sole option in the majority of cases^3,21,25^. In patients with ulcerated or bleeding tumours, a short course of intense radiotherapy may confer palliative benefit^26^. Alternatively, hyperthermia, hypoxia, chemotherapy or radiation can be applied in synergistic approaches including hyperthermic isolated limb perfusion, isolated limb infusion, and regional hyperthermia plus radiotherapy or chemotherapy^27–29^. In the past, local immunotherapy with interleukin-2 has been used sporadically, achieving CRRs exceeding 60% in melanoma^30^. Finally, also ablative therapies lend themselves to the treatment of soft tissue metastases. These interventional radiology procedures include radiofrequency, microwave and cryoablation, as well as arterial embolisation and IRE^4,5,31,32^. These techniques greatly vary in complexity and fundamental principles, and should only be applied within a shared multidisciplinary algorithm^33^.

VEG-ECT may represent an attractive option for surgical oncologists and interventional radiologists, with some potential merits. First, tissue preservation. Being a non-thermal ablative modality, it relies on reversible electroporation and a selective cytotoxic effect on replicating cells. Its safety in bone and liver tissue is documented in animal models and human studies^13,16,34,35^. A second advantage includes the coverage of large target volumes. Indicatively, a 5-electrode array (four peripheral electrodes in a 3-cm square fashion with a centrally placed electrode) with a 4-cm active tip is capable of targeting a 36-cm^3^ tissue volume. Third, the possibility to refine the ablation zone by selectively placing additional electrode pairs. Importantly, this enables VEG-ECT to achieve precise tumour coverage and include adequate safety margins while respecting surrounding tissues. Finally, repeatability and combination with *standard* ECT within the same procedure offer the unique opportunity to treat tumours of different size and depth within the same procedure. This combination is particularly advantageous in patients with melanoma or sarcomas who present with soft tissue metastases, which are heterogeneous in size and anatomical location and often are scattered.

At the same time, the limitations of this approach must be acknowledged. First, VEG-ECT entails the application of multiple electrodes, which could make the procedure cumbersome and time-consuming in some patients. This, nevertheless, soon could be overcome by the adoption of expandable catheters or more sophisticated tools for treatment planning^14^. Another concern relates to the insertion of relatively large probes and delivery of high voltages, which makes VEG-ECT more invasive and, conceivably, less tolerable compared to *standard* ECT. As a result, clinicians should adjust the anesthesiological management. In our experience, however, mild general or spinal anaesthesia without neuromuscular-blocking drugs ensured patient comfort and prevented anatomic motion during the procedure. Of note, the direct intense muscle stimulation produced by electric pulses may displace electrode probes and compromise treatment application. Adequate tumour coverage with electric fields requires not only accurate electrode insertion^12,36^ but also their stability during the phase of pulse delivery, which generally lasts some tens of seconds. Finally, treatment planning, image-guidance, individual electrode placement, and coordination with the software of the pulse generator inevitably add some complexity to the procedure. Nevertheless, by determining the best approach and electrode configuration, these are fundamental aspects of VEG-ECT technique and result particularly helpful when dealing with tumours located in challenging anatomical sites.

Interestingly, different supporting tools and navigation systems have been proposed to add precision and safety to VEG-ECT. These advancements will be particularly helpful in the treatment of intra-abdominal tumours such as liver malignancies and pancreatic cancer, or some forms of head and neck cancer^14^. As to soft tissue malignancies, according to our experience, an accurate and safe electrode placement can be achieved through US guidance, at least in patients with suprafascial lesions, while CT assistance can be reserved for selected patients with intramuscular lesions.

Our results have the obvious limitations of a single-arm phase 2 design and include its small scale, patient heterogeneity, the absence of a control group and independent radiological review, the novelty of the procedure, which inevitably implied a learning phase as in other therapies^32^ and on-study technique modifications. Admittedly, other variables might have influenced treatment outcome, including distribution of bleomycin and electric current within inhomogeneous tumour tissues^37^, and a discrepancy between pre-treatment plan and actual electrode placement. On this regard, a preliminary experience in patients with liver metastases suggests that US scan holds promise for real-time assessment of the electroporation process^38^, while experimental evidence in animal models indicates that magnetic resonance electrical impedance tomography (MREIT) may be a reliable instrument to predict electric field distribution^39^. Conversely, PET-CT scan performed at one month confirmed the strong antitumour effect of ECT but provided no additional predictive information regarding final treatment outcome; therefore, we believe that a single radiological evaluation may be appropriate to evaluate VEG-ECT response in routine clinical practice.

Finally, the availability of a highly active and safe locoregional therapy is particularly intriguing in the light of the proliferation of new targeted therapies and immunotherapies^14,40^, which have improved patient outcome and are opening new avenues for synergistic approaches aimed at consolidating local disease control and prolonging patient survival^6,7,41^.

Collectively, our data provide evidence that the novel ECT approach is feasible and safe in patients with locally advanced or metastatic tumours of the soft tissue. A single course of treatment led to complete resolution in 60% of cases, with low complications. These outcomes support VEG-ECT as a promising locoregional therapy for patients with bulky or deep-seated soft tissue metastases not amenable to surgical resection. Improvement of treatment planning and image guidance, real-time assessment of tissue electroporation, and further technical evolution have the potential to streamline the procedure. Additional detailed investigations are warranted to standardise the technique, define selection criteria and incorporate VEG-ECT into novel effective strategies. Our results highlight the benefit of VEG-ECT, advance the state-of-the-art in this field and lay the groundwork on which to plan future studies.

## Supplementary information

Supplementary Information.

Supplementary Information2.

## Data Availability

The individual patient data set is available upon reasonable request from the corresponding author.
