# Supplementary material for: Ablation of soft tissue tumours by long needle variable electrode-geometry electrochemotherapy: final report from a single-arm, single-centre phase-2 study
Source: Sci Rep. 2020 Feb 10;10:2291. doi: 10.1038/s41598-020-59230-w (PMC7010705; doi:10.1038/s41598-020-59230-w)
Supplement: Supplementary file 1 — Supplementary Information. [file 41598_2020_59230_MOESM1_ESM.pdf]

Scientific Reports

SUPPLEMENTARY METHODS

Ablation of soft tissue tumours by long needle variable electrode-geometry electrochemotherapy: final report from a single-arm, single-centre phase 2 study

Andrea Simioni[1*] Sara Valpione[2,3*], Elisa Granziera[4], Carlo Riccardo Rossi[5], Francesco Cavallin[6], Romina Spina[4], Elisabetta Sieni[7,8], Camillo Aliberti[9], Roberto Stramare[10], Luca Giovanni Campana[5*]

[1] University of Padova School of Medicine and Surgery, Padova, Italy

[2] The Christie NHS Foundation Trust, Manchester, UK

[3] Cancer Research UK Manchester Institute, The University of Manchester, Manchester, UK

[4] Veneto Institute of Oncology IOV-IRCCS, Padova, Italy

[5] Department of Surgical Oncological and Gastroenterological Sciences DISCOG, University of Padova, Padova, Italy

[6] Independent statistician, Solagna, Italy

[7] Department of Industrial Engineering, University of Padova, Padova, Italy

[8] Insubria University, Department of Theoretical and Applied Sciences – DiSTA, Varese, Italy

[9] Radiology Unit, Azienda Ospedaliera di Padova, Padova, Italy

[10] Radiology Unit, Department of Medicine DIMED, University of Padova, Italy

* These authors contributed equally

Supplementary Methods S1

Università degli Studi di Padova

DIPARTIMENTO DI SCIENZE ONCOLOGICHE E CHIRURGICHE

CLINICA CHIRURGICA II

Responsabile: Prof. Donato Nitti

Policlinico Universitario, via Giustiniani, 2 - 35128 Padova

Padova, 13/03/2009

Protocollo: **Studio pilota sull'elettrochemioterapia con bleomicina per il trattamento di tumori dei tessuti molli profondi o di grandi dimensioni (3-7 cm)**

Introduzione

Elettrochemioterapia

L'elettroporazione è un metodo fisico per aumentare il trasporto di farmaci, geni, o sonde molecolari all'interno delle cellule, basato sull'applicazione di campi elettrici che aumentano la permeabilità della membrana cellulare (1). L'elettrochemioterapia (ECT) è un efficace trattamento palliativo basato sull'elettroporazione delle cellule tumorali che è entrato nella pratica dell'oncologia clinica. Esso è rivolto a pazienti con noduli tumorali cutanei e sottocutanei, e sfrutta l'azione sinergica di brevi impulsi elettrici applicati localmente (elettroporazione reversibile) e di agenti antitumorali caratterizzati da bassa permeabilità (1,2). Tra i numerosi farmaci testati in studi preclinici, la bleomicina e il cisplatino sono risultati i più adatti per l'ECT. Questo approccio terapeutico garantisce un elevato tasso di risposta locale pur con bassi dosaggi di farmaco chemioterapico così da non causare rilevanti effetti collaterali (3). Inoltre, quest'approccio terapeutico garantisce la preservazione del tessuto normale grazie alle caratteristiche fisiche intrinseche del tessuto tumorale stesso e alla conformazione geometrica degli elettrodi che sono applicati solamente in corrispondenza delle lesioni tumorali da trattare.

In seguito al suo sviluppo sperimentale all'Istituto Gustave Roussy di Parigi nei primi anni novanta, l'ECT è entrata rapidamente in ambio clinico. Il progetto europeo ESOPE (European Standard Operating Procedures on the electrochemotherapy) ha favorito la standardizzazione di questo trattamento, provvedendo a fornire le informazioni necessarie per la diffusione dell'uso dell'ECT nella pratica clinica (4-6). Lo studio ESOPE ha reclutato 41 pazienti con noduli cutanei e sottocutanei minori di 3 cm. L'ECT si è dimostrata efficace sia nel trattamento di metastasi in transito da melanoma che in metastasi superficiali originate da altri tipi di tumore (sia cutanei, che viscerali), con

una risposta completa nel 74% dei noduli trattati, parziale nell'11%, nessuna variazione dimensionale nel 10% (secondo i criteri WHO); il controllo locale di malattia è risultato del 73-88% a 5 mesi dal trattamento.

Nella nostra iniziale esperienza clinica, iniziata nel 2006, abbiamo trattato oltre 50 pazienti affetti da metastasi superficiali originate da vari istotipi tumorali, in prevalenza melanoma e carcinoma della mammella, che non erano suscettibili al trattamento con le terapie convenzionali (chirurgia, radioterapia, per fusione d'arto, chemioterapia, immunoterapia). Sono stati trattati in totale 608 noduli tumorali (media 12 / pz); il 27% dei pazienti era affetto da noduli maggiori di 3 cm. La tollerabilità al trattamento è stata buona, specialmente in sedazione profonda. Una risposta oggettiva è stata ottenuta 50 dei 52 pazienti (96%) un mese dopo la prima applicazione. Ventidue pazienti sono stati sottoposti ad un secondo trattamento (a causa di una risposta parziale o della comparsa di nuove lesioni al di fuori del campo di trattamento). I pazienti parzialmente responsivi al primo trattamento hanno ottenuto un consolidamento della risposta: 80% di risposte complete, 20% di risposte parziali. Alcuni pazienti hanno ricevuto più di 5 trattamenti per la comparsa di nuove lesioni, ma hanno mantenuto il controllo superficiale di malattia. Dopo un follow-up medio di 13 mesi (range 4-29), solo 2 pazienti hanno sviluppato una recidiva locale. Attraverso un questionario appositamente creato di otto domande (riguardanti il sanguinamento locale delle lesioni tumorali, il loro impatto sull'aspetto estetico, sulle attività della vita quotidiana, le relazioni sociali, il dolore, la soddisfazione globale del trattamento, il consenso all'eventuale ritrattamento), la maggior parte dei pazienti ha riportato un miglioramento per quanto riguarda i disagio correlati alla malattia ed un beneficio nello svolgimento delle attività della vita quotidiana, ottenendo così un miglioramento della qualità di vita (7). Numerosi si sono rivelati i benefici legati all'impiego dell'ECT in ambito clinico dopo la standardizzazione della procedura (7): un'alta percentuale di risposta locale al trattamento, preservazione dei tessuti sani, breve ospedalizzazione, ripetibilità del trattamento e favorevole rapporto costo-beneficio rispetto ad altre possibilità terapeutiche. Finora l'impiego dell'ECT è stato limitato ai tumori di piccole dimensioni localizzati nel tessuto cutaneo e sottocutaneo; la strumentazione attuale (elettrodi di lunghezza massima di 3 cm), infatti, non ne permette l'applicazione alle lesioni profonde o di grandi dimensioni (maggiori di 3 cm). L'esperienza clinica ha tuttavia dimostrato che l'ECT può efficacemente distruggere anche tumori più estesi di 3 cm, anche se questo richiede in genere multiple applicazioni e di conseguenza più sedute terapeutiche per il paziente.

Razionale dello studio

La possibile spiegazione dei casi di mancata risposta al trattamento con ECT potrebbe risiedere negli aspetti tecnici della metodica (applicazione degli impulsi elettrici in corrispondenza del tessuto bersaglio) o nella resistenza intrinseca del tumore al farmaco impiegato. Nella nostra esperienza, i più rilevanti ostacoli sono stati i seguanti: 1) tumori estesi che coinvolgono vaste aree anatomiche, che richiedono in genere ripetute applicazioni degli elettrodi nella stessa seduta che risultano dispendiose in termini di tempo (considerando che la finestra di tempo utile per l'applicazione delle correnti elettriche dopo l'iniezione del farmaco è di soli 20 minuti secondo le procedure standard); 2) tumori di grandi dimensioni (>3 cm), a causa dell'impossibilità tecnica di raggiungere la parte più profonda del tumore alla prima applicazione con gli elettrodi attualmente disponibili; 3) profondità del tumore (>3 cm); 4) campi precedentemente irradiati, per la parziale penetrazione dell'ago dell'elettrodo e la diffusione subottimale della corrente elettrica nel tessuto fibroso (7). La disponibilità di nuovi strumenti tecnici (elettrodi, hardware) potrebbe espandere le indicazioni dell'ECT, portando al trattamento di noduli tumorali dei tessuti molli più grandi e più profondi. Una recente revisione di 118 pazienti con metastasi di tessuti molli (coinvolgenti muscolo scheletrico o tessuto sottocutaneo) afferenti ad un singolo centro di ricerca negli USA, ha infatti rilevato che i pazienti con noduli > di 3 cm erano oltre un terzo (38/118, 32%) (8). Il miglioramento dell'ECT come opzione terapeutica per i tumori dei tessuti molli profondi o di grandi dimensioni – primitivi o metastatici - potrebbe costituire un'alternativa terapeutica molto efficace e poco invasiva, garantendo così un beneficio per i pazienti che dovrebbero altrimenti affrontare un intervento chirurgico in genere gravoso con conseguente peggioramento della qualità di vita e riduzione del loro performance status (8-10).

Ipotesi di lavoro

Attualmente l'erogazione di impulsi elettrici al tumore avviene per mezzo di manipoli dotati di un numero fisso di elettrodi ad ago (di lunghezza massima pari a 2-3 cm) collocati ad una distanza fissa tra di loro. La disponibilità di elettrodi più lunghi e posizionabili in maniera indipendente l'uno dall'altro così da ottenere una configurazione geometrica flessibile (adattabile alla singola lesione da trattare) può generare un campo elettrico più ampio e omogeneo, capace di coprire tutto il bersaglio tumorale ed i suoi margini, ottenendo così una migliore risposta ed un controllo di malattia anche nei tumori delle parti molli profondi e/o grandi, con un'unica seduta di ECT mantenendo una tossicità locale modesta, sovrapponibile a quella riscontrata con l'applicazione degli elettrodi attualmente in uso. Un tale risultato permetterebbe di evitare al paziente la necessità di sottoporsi a ripetute sedute

di ECT oppure di sottoporsi ad interventi chirurgici complessi, demolitivi, e pertanto associati ad una non trascurabile morbilità.

Scopo dello studio

L'obbiettivo principale di questo studio è quello di valutare l'attività (risposta tumorale a 2 mesi) dell'ECT applicata per mezzo di un nuovo generatore d'impulsi elettrici a "geometria variabile" (Cliniporator-VG) e di nuovi elettrodi. Inoltre, saranno valutati gli aspetti tecnici e la tossicità locale. Eventuali problematiche tecniche dovute all'impiego della nova strumentazione saranno valutate dopo ogni singolo paziente. Saranno inoltre valutate la tossicità locale, la durata della risposta locale (in caso di assenza di ulteriori trattamenti neoplastici nel corso del follow-up), la qualità di vita e la distribuzione del farmaco nel sangue periferico e nel tumore.

<u>Metodi</u>

Disegno dello studio

Questo studio sarà condotto utilizzando un disegno "single-stage" secondo Fleming. L'efficacia dell'ECT sarà valutata in termini di risposta antitumorale locale. Se il tasso di risposta locale sarà inferiore o uguale al 40%, esso sarà ritenuto insufficiente per proseguire successive valutazioni della metodica. Invece, un tasso di risposte complete >60% sarà giudicato clinicamente sufficiente e sarà un'indicazione a nuovi studi. Questo livello di efficacia è stato scelto in base alla nostra precedente esperienza con l'ECT in pazienti con piccole lesioni e considerando i dati della letteratura (3-4,6,7). Con un campione di 30 pazienti, il rischio di raccomandare erroneamente una procedura con tasso di risposte complete inadeguato è del 5%, mentre la possibilità di rifiutare erronemanete il trattamento è minore al 20% nel caso in cui il tasso di risposta completa atteso della metodica è del 60%.

Considerazione statistiche

L'analisi principale sarà effettuata su tutti i pazienti eleggibili sottoposti ad almeno una seduta di elettro-chemioterapia. Per la valutazione dell'obbiettivo principale, si fornirà la stima della percentuale di risposta obbiettive locale con i relativi limiti di confidenza al 95% calcolati con il metodo esatto. La percentuale di remissioni sarà determinato considerando il numero di pazienti che raggiungono la risposta dopo trattamento sul numero di pazienti totali di cui viene rivalutato lo stato di malattia dopo il trattamento stesso. La sopravvivenza e la durata della risposta locale saranno calcolate dalla data del trattamento con ECT e descritte mediante il metodo di Kaplan-Meier. La

tossicità sarà decritta mediante tabelle di frequenza riportando il grado massimo per paziente per i diversi sottotipi di tossicità.

Selezione dei pazienti

I pazienti saranno consecutivamente arruolati tra quelli affetti da tumori dei tessuti molli (primitivi o metastatici) istologicamente documentati, di qualsiasi tipo (al massimo una lesione compresa tra 3 e 7 cm o profonda più di 3 cm fino ad un massimo di 20 cm), non suscettibile di trattamento con le terapie convenzionali (chirurgia, radioterapia, chemioterapia) come discusso collegialmente nell'ambito dei meeting multidisciplinari (Gruppo Melanoma, Gruppo Tumori dei Tessuti Molli). Una massa tumorale con le caratteristiche sopraddette sarà selezionata come lesione bersaglio (*target lesion*) e trattata con ECT applicata per mezzo delle nuove strumentazioni (Cliniporator-VG ed elettrodi dedicati). La massa tumorale bersaglio sarà misurata con un calibro se superficiale, o attraverso risonanza magnetica, tomografia assiale computerizzata o ecografia; in tale occasione sarà esclusa da un radiologo dedicato la vicinanza della lesione a vasi o nervi, tale da precludere un inserimento sicuro degli elettrodi per via percutanea al momento del trattamento. In caso di multiple lesioni con le caratteristiche richieste, la scelta della lesione target, avverrà tenendo conto della sede anatomica (privilegiando sedi in cui l'applicazione degli elettrodi sia tecnicamente più agevole e sicura come il dorso o gli arti) e della sintomaticità delle lesioni stesse come riferito dal paziente (lesioni sanguinanti, dolenti, o inficianti attività di vita quotidiana, ecc.). Saranno inoltre registrati l'istologia del tumore, la regione anatomica da trattare ed i precedenti trattamenti effettuati in apposite schede di raccolta dati. La presenza e l'estensione di altre localizzazioni tumorali sarà valutata con esame clinico e tecniche radiologiche di imaging standard.

Criteri di inclusione:

- Pazienti con noduli tumorali localizzati nei tessuti molli misurabili e suscettibili di applicazione degli elettrodi
- Performance status ≤ 2 secondo la scala ECOG
- Dimensioni del tumore comprese fra 3 e 7 cm o di profondità > 3 cm
- Consenso informato

Criteri di esclusione:

- Grave patologia cardiaca polmonare o epatica
- Epilessia
- Portatori di pace-maker (per lesioni della parete toracica)
- Spettanza di vita < 3 mesi

- Infezione in atto
- Precedenti trattamenti con bleomicina fino alla dose massima totale
- Altri trattamenti antitumorali somministrati nelle 4 settimane precedenti l'ECT e nelle 8 settimane successive
- Alterazioni della coagulazione del sangue
- Lesioni tumorali non adatte all'inserimento degli elettrodi (poste in prossimità a vasi o tronchi nervosi)

Valutazione pre-operatoria

I pazienti saranno valutati attraverso un esame clinico, esami ematologici, ECG e radiografia del torace. Tutti i pazienti saranno studiati radiologicamente a con TAC od RMN ed ecografia dei tessuti molli pre-operatoria della lesione bersaglio per misurarne le dimensioni e la profondità. La stadiazione avverrà con TC total-body e TC-PET. Saranno escluse lesioni vicine a grossi vasi o tronchi nervosi. La distribuzione del campo elettrico attorno al tumore sarà oggetto di una pianificazione prima del trattamento in modo da scegliere le modalità operative (inserimento degli elettrodi) con cui condurre la terapia, così da assicurare che la ampiezza del campo elettrico sia sufficiente ad ottenere l'elettroporazione della membrana cellulare. Inoltre, il piano pre-trattamento sarà volto a rendere il numero di elettrodi da inserire il minimo possibile, cosicché la procedura sia semplice e meno invasiva. Tutti i pazienti saranno inoltre sottoposti ad una visita anestesiologica prima del trattamento.

Strumentazione: generatore di impulsi elettrici (Cliniporator-VG)

Il dispositivo medico Cliniporator-VG (Variable Geometry) è uno strumento per l'elettroporazione tissutale, ideato e prodotto da IGEA S.p.A. (Carpi, Italia). Tutti I dispositivi per l'elettroporazione tissutale prodotti da IGEA, come anche Cliniporator-VG sono certificati dalla CE per il loro utilizzo nella pratica clinica (allegato-1). Il Cliniporator™ è attualmente utilizzato in più di 20 centri in Italia e altri 20 in Europa. La più importante applicazione dell'elettroporazione è il trattamento dei tumori della pelle e delle metastasi cutanee e sottocutanee. Questo studio propone l'utilizzo del Cliniporator-VG, una migliorata versione del Cliniporator™, per il trattamento di tumori ampi o profondi. Il Cliniporator-VG è in grado di trattare volumi di tessuto grandi (più di 10 cm cubi) e utilizza 6 elettrodi indipendenti che possono essere posizionati liberamente per trattare completamente il tumore, indifferentemente dalla profondità e dimensione.

Strumentazione: nuovi elettrodi ad ago singolo

Gli elettrodi ad ago singolo sono stati sviluppati per il trattamento di grandi tumori dei tessuti molli per via percutanea. Questi elettrodi sono certificati per uso clinico (allegato-2) composti da uno stelo metallico lungo 20 cm parzialmente isolato e sono provvisti come dispositivi sterili per un singolo utilizzo. Il corpo centrale dell'elettrodo è ricoperto da uno strato isolante in modo che l'applicazione del campo elettrico sia limitato alla regione terminale "attiva" e sia preservato il tessuto sano che circonda la lesione.

Per garantire un trattamento centrato sulla specifica profondità e dimensione della singola lesione, sono disponibili elettrodi con differenti caratteristiche:

- Diametro: 1.2 o 1.8 mm
- Segmento "attivo" (non isolato) compreso fra 2 e 5 cm
- Lunghezza complessiva: 20 cm.

Trattamento

I pazienti affetti da noduli sottocutanei multipli riceveranno l'ECT standard secondo le linee guida dell'ESOPE e con elettrodi standard, durante la stessa sessione terapeutica. La lesione bersaglio oggetto del presente studio, invece, sarà sottoposta ad elettroporazione attraverso l'inserzione eco-guidata dei nuovi elettrodi, connessi al nuovo generatore di impulsi elettrici (Clinipoator-VG).

Il trattamento prevede due fasi: la somministrazione della bleomicina per via endovenosa (alla dose di 15.000 UI /m^2) in bolo e quindi l'erogazione degli impulsi elettrici. Tali impulsi, applicati durante il trattamento della lesione bersaglio, avranno gli stessi parametri degli impulsi elettrici utilizzati nello studio ESOPE e successivamente diffusi nella pratica clinica: una serie di 8 impulsi di 100 microsecondi, con ampiezza tale da generare un campo elettrico locale di 100 volt (V)/cm. Gli elettrodi saranno posizionati da un radiologo sotto guida ecografica con il paziente in sedazione od anestesia generale a seconda del giudizio dell'anestesista. L'inserimento degli elettrodi avverrà con l'aiuto di un supporto rigido (template) che ha lo scopo di mantenere una minima distanza efficace tra gli elettrodi stessi pur consentendone una disposizione variabile in considerazione della specifica situazione clinica (geometria e situazione della massa tumorale).

Studio di farmacocinetica

Saranno misurati i livelli plasmatici di bleomicina in campioni ematici prelevati a 8 e 28 minuti dopo la somministrazione endovena del farmaco. Saranno inoltre misurati i livelli di bleomicina nel tessuto tumorale mediante cromatografia (HPLC) in campioni di tessuto ottenuti per mezzo di una

microbiopsia con ago tranciante (tru-cut) 8 minuti dopo l'infusione di BLM (immediatamente prima dell'elettroporazione).

Valutazione della risposta tumorale e follow-up

Dopo ogni procedura saranno valutati i problemi tecnici riscontrati nel posizionamento degli elettrodi e la tossicità locale. La tossicità locale e sistemica verrà inoltra valutata con esame fisico, esami ematochimici ed eventuale ecografia in prima giornata post-trattamento ed a 2, 4, 8, 12 e 16 settimane e quindi secondo il follow-up standard concordato con l'oncologo di riferimento (3-6 mesi).
La risposta tumorale sarà definita radiologicamente con TAC od RMN (a seconda dell'esame di imaging eseguito prima del trattamento) seguendo i criteri RECIST (13) a 30 e 60 giorni dal trattamento. La parziale risposta è definita come una riduzione ≥ del 50% della soma del prodotto dei diametri massimi perpendicolari della lesione bersaglio dopo almeno 1 mese; la risposta completa è definita come la scomparsa della massa tumorale dopo almeno 1 mese. I pazienti che non incontrano i criteri per la risposta parziale o completa saranno definiti non responsivi. Nel caso in cui il paziente sia considerato "libero da malattia" con l'asportazione del tumore, gli sarà offerta la possibilità dell'asportazione chirurgica dello stesso: la lesione asportata verrà sottoposta ad esame istologico. I pazienti che avranno una risposta parziale e che saranno sottoposti, se tecnicamente possibile, ad altri trattamenti locali (asportazione chirurgica o seconda ECT) saranno giudicati come "partial responders" fino alla data del ritrattamento. La sopravvivenza e la durata della risposta locale saranno calcolate dalla data del trattamento con ECT.

Valutazione della tossicità e qualità di vita

La tossicità locale e sistemica del trattamento sarà valutata mediante esame clinico in occasione delle visite di controllo e registrata secondo i "Common Toxicity Criteria" (Common Terminology Criteria for Adverse Events, CTCAE, versione 3.0). I pazienti eseguiranno un prelievo per valutazione dell'emocromo e della funzionalità epatica e renale dopo 7 e 15 giorni dal trattamento.
L'impatto del trattamento sulla qualità di vita sarà studiato mediante apposito questionario validato (EORTC QLQ-C30, Euro QoL) e mediante alcune semplici domande che indagano le condizioni locali della cute che è sede delle lesioni tumorali trattate. Tali questionari saranno somministrati prima del trattamento ed alle visite di controllo (30, 60 e 90 giorni).

Bibliografia

1. Belehradek M, Domenge C, Luboinski B, et al. Electrochemotherapy, a new antitumor treatment. First clinical phase I—II trial. *Cancer* 1993; 72:3694–700.

2. Mir LM, Orlowski S. Mechanisms of electrochemotherapy. *Adv Drug Del Rev*1999; 35:107–118.

3. Gothelf A, Mir LM, Gehl J. Electrochemotherapy: results of cancer treatment using enhanced delivery of bleomycin by electroporation. *Cancer Treat Rev* 2003; 29:371–387.

4. Serša G. The state-of-the-art of electrochemotherapy before the ESOPE study; advantages and clinical use. *EJC Suppl* 2006; 4:52–9.

5. Mir LM, Gehl J, Sersa G, et al. Standard operating procedures of the Electrochemotherapy: instructions for the use of bleomycin or cisplatin administered either systemically or locally and electric pulses delivered by the Cliniporator by means of invasive or non-invasive electrodes. *EJC Suppl* 2006; 4:14–25.

6. Marty M, Serša G, Garbay JR, et al. Electrochemotherapy—An easy, highly effective and safe treatment of cutaneous and subcutaneous metastases: results of ESOPE study. *EJC Suppl* 2006; 4:3–13.

7. Campana LG, Mocellin S, Basso M, et al. Bleomycin-based electrochemotherapy: clinical outcome from a single institution's experience with 52 patients. Ann Surg Oncol. 2009 Jan;16(1):191-9.

8. Damron TA, Heiner J. Distant soft tissue metastases: a series of 30 new patients and 91 cases from the literature. *Ann Surg Oncol* 2000; 7: 526-34

9. Vezeridis MP, Moore R, Karakousis CP. Metastatic patterns in soft tissue sarcomas. *Arch Surg* 1983; 118: 915-18

10. Huth JF, Eilber FR. Patterns of metastatic spread following resection of extremity soft tissue sarcomas and strategies for treatment. Semin Oncol 1998; 4: 20-26

11. Mir LM, Glass LF, Sersa G, et al. Effective treatment of cutaneous and subcutaneous malignant tumours by electrochemotherapy. *Br J Cancer*. 1998 Jun;77(12):2336–42.

12. Byrne MC, Thompson JF, Johnston H, et al. Treatment of metastatic melanoma using electroporation therapy with bleomycin (electrochemotherapy). *Melanoma Res* 2005;15:45–51.

13.. Therasse P, Arbuck SG, Eisenhauer EA. New guidelines to evaluate the response to treatment in solid tumors. European Organization for Research and Treatment of Cancer, National Cancer Institute of the United States, National Cancer Institute of Canada. *J Natl Cancer Inst*. 2000;92:205–16.

Allegati

Allegato-1

CERTIFICATO CE

Certificato n. 1178/MDD

Dichiarazione di approvazione del sistema qualità

(Sistema completo di garanzia qualità)

Visto l'esito delle verifiche condotte in conformità all'Allegato II, punto 3 del Decreto Legislativo 24 febbraio 1997, n. 46, attuazione della direttiva 93/42/CEE, si dichiara che la ditta:

IGEA SPA

mantiene negli stabilimenti di:

CARPI (MO) - VIA PARMENIDE 10/A (ITA) - Italy

un sistema qualità che assicura la conformità dei seguenti prodotti:

Apparecchio per elettroporazione e relativi accessori
Mod. VGP01.
Marca IGEA

Mod. CAVG (Accessorio - cavo adattatore per elettroporatore serie VGP)
Mod. VGC-01(Accessorio - cavo sterilizzabile per elettroporatore serie VGP)

ai requisiti essenziali ad essi applicabili del Decreto suddetto, in tutte le fasi dalla progettazione al controllo finale.

Riferimento pratiche IMQ: 10AI00220; 10AI00057; 10AJ00063.

Questa Dichiarazione di approvazione è rilasciata dall'IMQ S.p.A. quale organismo notificato per la direttiva 93/42/CEE.
Il numero identificativo dell'IMQ S.p.A. quale organismo notificato è: 0051.

Emesso il: 2008-11-21
Data di Aggiornamento: 2009-05-05
Sostituisce: 2008-11-21

IMQ

Questa Dichiarazione di approvazione è soggetta alle condizioni previste dall'IMQ nel "Regolamento per la certificazione CE dei dispositivi medici in base alla direttiva 93/42/CEE".
Essa non è comunque valida dopo il 2013-11-20 (articolo 11, comma 11 della direttiva).

EC CERTIFICATE

Certificate No 1178/MDD

Full Quality Assurance System Approval Certificate

On the basis of our examination carried out according to Annex II, section 3 of Legislative Decree of 1997-02-24, No 46, national transposition of the Directive 93/42/EEC, we hereby certify that:

IGEA SPA

manages in the factories of:

CARPI (MO) - VIA PARMENIDE 10/A (ITA) - Italy

a full quality assurance system ensuring the conformity of the following products:

Electroporation devices and related accessories
Type ref. VGP01.
Trade mark IGEA

Type ref. CAVG (Accessorie - cable adaptor for electroporator series VGP)

Type ref. VGC-01 (Accessorie - sterilizable cable for electroporator series VGP)

with the relevant essential requirements of the aforementioned national legislation transposing the Directive 93/42/EEC, from design to final inspection and testing.

Reference to IMQ files Nos: 10AI00220; 10AI00057; 10AJ00063.

This Approval Certificate is issued by IMQ S.p.A. as Notified Body for the Directive 93/42/EEC.
Notified Body notified to European Commission under number: 0051.

Date:	2008-11-21
Updated:	2009-05-05
Substitution Date:	2008-11-21

IMQ

This Approval Certificate is subjected to the provisions laid down in the "Rules for managing the EC Certification of Medical Devices on the basis of the Directive 93/42/EEC".
In any case, it does not remain valid after 2013-11-20 (article 11, clause 11 of the Directive).

This is a translation of the Italian text, which prevails in case of doubts

Allegato-2

CERTIFICATO CE

Certificato n. 1217/MDD

Dichiarazione di approvazione del sistema qualità

(Sistema completo di garanzia qualità)

Visto l'esito delle verifiche condotte in conformità all'Allegato II, punto 3 del Decreto Legislativo 24 febbraio 1997, n. 46, attuazione della direttiva 93/42/CEE, si dichiara che la ditta:

IGEA SPA

mantiene negli stabilimenti di:

CARPI (MO) - VIA PARMENIDE 10/A (ITA) - Italy

un sistema qualità che assicura la conformità dei seguenti prodotti:

Elettrodi monouso sterili per elettroporatore serie VGP
Modd. VG12-30; VG12-40; VG18-30; VG18-40.
Marca IGEA

ai requisiti essenziali ad essi applicabili del Decreto suddetto, in tutte le fasi dalla progettazione al controllo finale.

Riferimento pratiche IMQ: 10AJ00025.

Questa Dichiarazione di approvazione è rilasciata dall'IMQ S.p.A. quale organismo notificato per la direttiva 93/42/CEE.
Il numero identificativo dell'IMQ S.p.A. quale organismo notificato è: 0051.

Emesso il: 2009-05-05

IMQ

Questa Dichiarazione di approvazione è soggetta alle condizioni previste dall'IMQ nel "Regolamento per la certificazione CE dei dispositivi medici in base alla direttiva 93/42/CEE".
Essa non è comunque valida dopo il 2014-05-04 (articolo 11, comma 11 della direttiva).
