# Supplementary material for: Ablation of soft tissue tumours by long needle variable electrode-geometry electrochemotherapy: final report from a single-arm, single-centre phase-2 study
Source: Sci Rep. 2020 Feb 10;10:2291. doi: 10.1038/s41598-020-59230-w (PMC7010705; doi:10.1038/s41598-020-59230-w)
Supplement: Supplementary file 2 — Supplementary Information2. [file 41598_2020_59230_MOESM2_ESM.pdf]

Scientific Reports

SUPPLEMENTARY MATERIAL

# Ablation of soft tissue tumours by long needle variable electrode-geometry electrochemotherapy: final report from a single-arm, single-centre phase 2 study

**Andrea Simioni[1*] Sara Valpione[2,3*], Elisa Granziera[4], Carlo Riccardo Rossi[5], Francesco Cavallin[6], Romina Spina[4], Elisabetta Sieni[7,8], Camillo Aliberti[9], Roberto Stramare[10], Luca Giovanni Campana[5*]**

[1] University of Padova School of Medicine and Surgery, Padova, Italy

[2] The Christie NHS Foundation Trust, Manchester, UK

[3] Cancer Research UK Manchester Institute, The University of Manchester, Manchester, UK

[4] Veneto Institute of Oncology IOV-IRCCS, Padova, Italy

[5] Department of Surgical Oncological and Gastroenterological Sciences DISCOG, University of Padova, Padova, Italy

[6] Independent statistician, Solagna, Italy

[7] Department of Industrial Engineering, University of Padova, Padova, Italy

[8] Insubria University, Department of Theoretical and Applied Sciences – DiSTA, Varese, Italy

[9] Radiology Unit, Azienda Ospedaliera di Padova, Padova, Italy

[10] Radiology Unit, Department of Medicine DIMED, University of Padova, Italy

* These authors contributed equally

**Supplementary Table S1.** Toxicity report (CTCAE v4.0)

| Toxicity | Time of assessment (No. of patients and %) | | | | | |
|---|---|---|---|---|---|---|
| | Baseline* | Post-ECT° | In-hospital | 1 month | 2 months | 6† months |
| <u>**Local**</u> | | | | | | |
| **Bleeding** | | | | | | |
| G1 | 7 (23) | 8 (27) | 3 (10) | 1 (3) | 0 | 0 |
| G2 | 1 (3) | 0 | 0 | 0 | 0 | 0 |
| **Oedema** | | | | | | |
| G1 | n.a. | 0 | 6 (20) | 3 (10) | 1 (3) | 0 |
| **Pain** | | | | | | |
| G1 | 12 (40) | 16 (53) | 8 (27) | 21 (70) | 10 (33) | 7 (17) |
| G2 | 4 (13) | 5 (17) | 1 (3) | 1 (3) | 3 (10) | 1 (3) |
| G3 | 0 | 0 | 0 | 1 (3) | 0 | 0 |
| **Sensory neuropathy** | | | | | | |
| G1 | 0 | 0 | 0 | 0 | 0 | 0 |
| G2 | 0 | 1 (3) | 1 (3) | 1 (3) | 1 (3) | 0 |
| **Skin/tumor ulceration** | | | | | | |
| G1 | 3(10) | 3 (10) | 3 (10) | 9 (30) | 11 (37) | 3 (10) |
| G2 | 4 (13) | 4 (13) | 4 (13) | 10 (33) | 5 (17) | 3 (10) |
| G3 | 4(13) | 4 (13) | 4 (13) | 2 (7) | 1 (3) | 1 (3) |
| **Skin/soft tissue infection** | | | | | | |
| **G**1 | 4 (13) | 3 (10) | 3 (10) | 2 (7) | 1 (3) | 2 (7) |
| G2 | 0 | 0 | 0 | 1 (3) | 5 (17) | 0 |
| G3 | 0 | 0 | 0 | 1 (3) | 1 (3) | 0 |
| **Soft tissue necrosis** | | | | | | |
| G2 | 0 | 0 | 0 | 1 (3) | 2 (7) | 0 |
| **Soft tissue fibrosis** | | | | | | |
| G1 | n.a. | 0 | 0 | 0 | 1 (3) | 1 (3) |
| G2 | | 0 | 0 | 0 | 2 (7) | 4 (13) |
| <u>**Systemic**</u> | | | | | | |
| **ARDS‡** | | | | | | |
| G5 | 0 | 0 | 0 | 0 | 0 | 1 (3) |
| **Colonic obstruction** | | | | | | |
| G1 | 0 | 0 | 0 | 1 (3) | 0 | 1 (3) |
| **Fever** | | | | | | |
| G1 | 0 | 0 | 5 (17) | 1 (3) | 0 | 2 (7) |
| G2 | 0 | 0 | 0 | 1 (3) | 1 (3) | 1 (3) |
| **Nausea / Vomiting** | | | | | | |
| G1 | n.a. | 2 (7) | 4 (13) | 1 (3) | 0 | 1 (3) |
| **Sinus bradycardia** | | | | | | |
| G1 | 0 | 0 | 1 (3) | 1 (3) | 0 | 0 |
| **Urinary tract infection** | | | | | | |
| G2 | 0 | n.a. | 1 (3) | 0 | 1 (3) | 0 |
| G3 | 0 | | 0 | 0 | 0 | 1 (3) |

*Legend*: ARDS, acute respiratory distress syndrome; CTCAE, Common Terminology Criteria for Adverse Events; ECT, electrochemotherapy;

* Seventeen patients were still receiving some forms of pain treatment at the time of enrollment

° Assessment performed in the recovery room, at the end of the anesthesiological procedure

† Toxicity assessment performed on 29 alive patients

‡ A 63-year-old female melanoma patient complained of progressively worsening respiratory symptoms starting from 16 weeks after ECT. Radiological imaging was suggestive of interstitial pneumonitis, and the patient was admitted in the intensive care unit and received respiratory support and full-dose steroid therapy but died one month later.

**Supplementary Table S2.** Postoperative pain according to patient characteristics and ECT parameters

| **Characteristics** | **1 month** | | **2 months** | |
|---|---|---|---|---|
| | **Pain grade*** | ***P*** | **Pain grade*** | ***P*** |
| **Histotype** | | 0.50 | | 0.14 |
| Melanoma | 1 (1-1) | | 0 (0-0) | |
| Sarcoma | 1 (0-1) | | 1 (0-1) | |
| **Target tumour location** | | 0.62 | | 0.95 |
| Upper limb | 1 (0-1) | | 0 (0-1) | |
| Trunk | 1 (0-1) | | 0 (0-1) | |
| Lower limb | 1 (1-1) | | 0 (0-1) | |
| **Target tumor size, mm** | 0.10 | 0.58 | 0.09 | 0.62 |
| **Tumour ulceration** | | 0.56 | | 0.28 |
| No | 1 (1-1) | | 0 (0-1) | |
| Yes | 1 (0-1) | | 1 (0-1) | |
| **Previous Tx on target tumour** | | 0.69 | | 0.11 |
| No | 1 (0-1) | | 0 (0-0) | |
| Yes | 1 (1-1) | | 1 (0-1) | |
| **Previous Tx on target tumour** | | 0.64 | | **0.005** |
| None or surgery | 1 (0-1) | | 0 (0-0) | |
| CT/ILP | 1 (1-1) | | 1 (1-1) | |
| **Synchronous visceral metastases** | | 0.72 | | 0.09 |
| No | 1 (1-1) | | 0 (0-1) | |
| Yes | 1 (1-1) | | 1 (0-1) | |
| **No. of electrode probes** | | 0.53 | | **0.04** |
| Five | 1 (0-1) | | 0 (0-0) | |
| Six | 1 (1-1) | | 1 (0-1) | |
| **No. of electrode placements°** | | **<0.001** | | 0.41 |
| 1 | 0 (0-0) | | 0 (0-0) | |
| ≥ 2 | 1 (1-1) | | 0 (0-1) | |
| **Electrode repositioning†** | | 0.90 | | 0.61 |
| No | 1 (0-1) | | 0 (0-1) | |
| Yes | 1 (1-1) | | 1 (0-1) | |
| **ECT duration** (min) | 0.26 | 0.17 | 0.22 | 0.24 |
| **Anaesthesia** | | 0.85 | | 0.99 |
| Spinal | 1 (1-1) | | 0 (0-0) | |
| General | 1 (1-1) | | 0 (0-1) | |

*Legend*: CT, chemotherapy; ILP, isolated limb perfusion
* Data expressed as n (%) or median (IQR)
° Number of placements of the whole electrode array to cover the target volume
† Patients in whom it was necessary to replace one or more probes due to low electric current

**Supplementary Table S3.** Local toxicity according to patient characteristics and ECT parameters

| Characteristic | One month | | Two months | |
|---|---|---|---|---|
| | Toxicity grade* | *P* | Toxicity grade* | *P* |
| **Histotype** | | | | |
| Melanoma | 1 (0-1) | **0.03** | 0 (0-1) | **0.03** |
| Sarcoma | 2 (1-2) | | 1 (0-1) | |
| **Target tumour location** | | | | |
| Upper limb | 1 (0-2) | 0.99 | 0 (0-1) | 0.81 |
| Trunk | 1 (1-2) | | 1 (0-1) | |
| Lower limb | 1 (1-2) | | 1 (0-1) | |
| **Tumor size** (mm) | 0.15 | 0.44 | 0.22 | 0.24 |
| **Tumour ulceration** | | | | |
| No | 1 (0-1) | **0.002** | 0 (0-1) | **0.001** |
| Yes | 2 (2-2) | | 1 (1-2) | |
| **Previous Tx on target tumour** | | | | |
| No | 1 (0-1) | 0.19 | 0 (0-1) | 0.51 |
| Yes | 1 (1-2) | | 1 (0-1) | |
| **Previous Tx on target tumour** | | | | |
| None or surgery | 1 (0-1) | 0.25 | 0 (0-1) | 0.24 |
| CT/ILP | 1 (1-2) | | 1 (0-1) | |
| **Synchronous visceral metastases** | | | | |
| No | 1 (1-2) | 0.98 | 1 (0-1) | 0.47 |
| Yes | 1 (0-2) | | 0 (0-1) | |
| **No. of electrode probes** | | | | |
| Five | 1 (0-1) | **0.03** | 0 (0-1) | 0.19 |
| Six | 2 (1-2) | | 1 (0-2) | |
| **No. of electrode placements°** | | | | |
| 1 | 0 (0-1) | 0.12 | 0 (0-0) | **0.04** |
| ≥ 2 | 1 (1-2) | | 1 (0-1) | |
| **Electrode repositioning**† | | | | |
| No | 1 (0-2) | 0.55 | 1 (0-1) | 0.56 |
| Yes | 1 (1-2) | | 1 (0-1) | |
| **ECT duration** (min) | 0.48 | **0.007** | 0.36 | **0.04** |

*Legend: CT, chemotherapy; ILP, isolated limb perfusion*

*Data expressed as median (IQR) or [a] Spearman rank correlation coefficient.

° Number of placements of the whole electrode array to cover the target volume

† Patients in whom it was necessary to replace one or more electrode probes due to low electric current

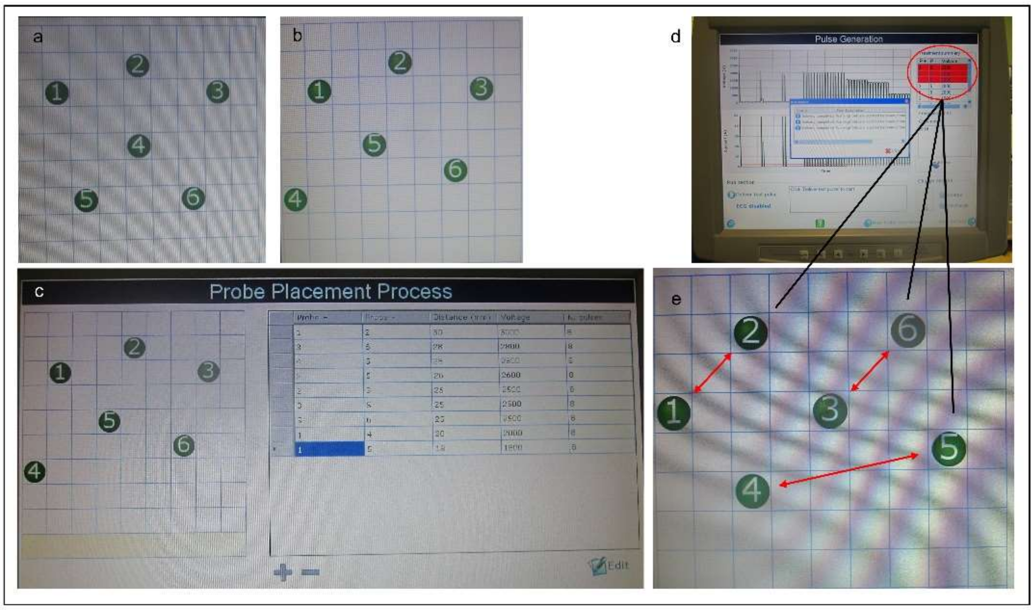


**Supplementary Figure S1.** Upload of electrode parameters (position and distances) into the software pulse generator. (**a,b**) The interface of the pulse generator with two configurations of the electrode array (its geometry is customised on tumour morphology). (**c**) Following probe placement, the actual distance between electrodes of each couple is uploaded into the generator to customise voltages. (**d,e**). Intraoperative treatment verification. Following pulse delivery, the software provides real-time feed-back by displaying the electric current recorded between each electrode pair, thus allowing for eventual adjustments. In this case, there is no current flow between three of the electrode pairs (red lines), prompting for their replacement to complete treatment.

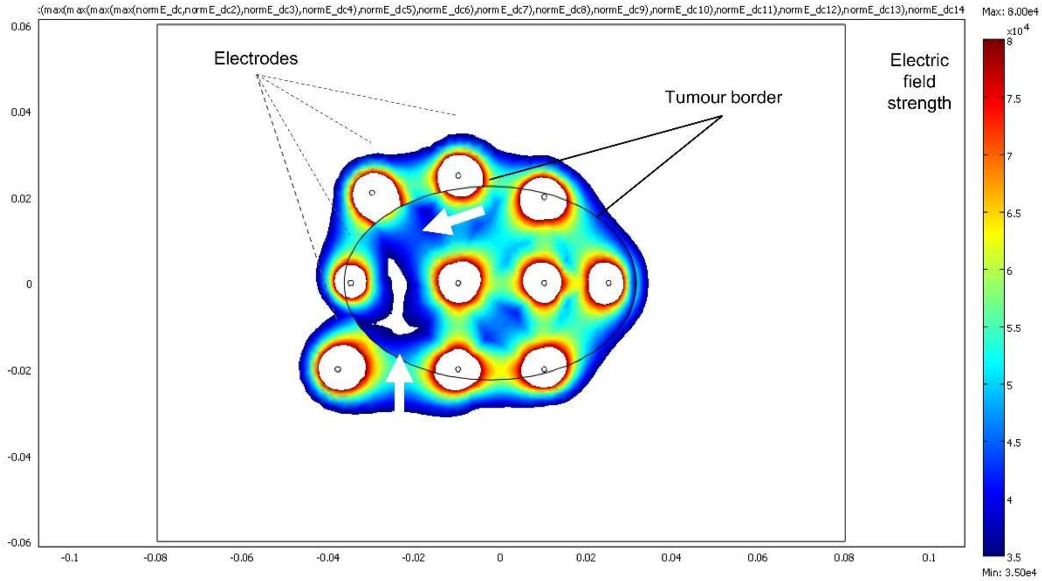


**Supplementary Figure S2.** Distribution of the electric field intensity around the tumour. The software of the pulse generator provides a treatment-specific map based on electrode disposition and intraoperative electric parameters, which can be used to evaluate the accuracy of treatment delivery. The heat map shows the electric field around a tumour treated using ten probes (the white arrows indicate an area of the target lesion which was not covered with electric fields).

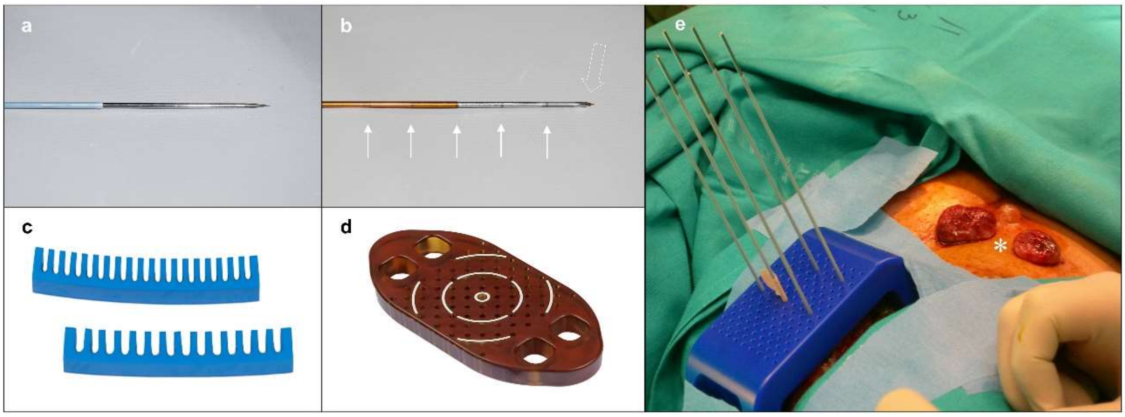


**Supplementary Figure S3.** Incremental improvement of VEG-ECT technique. Over the study, some modifications of the VEG-ECT technique were introduced through a dedicated audit process. (**a**) The initial version of the needle electrode with a "tapered shaped" design. (**b**) The newly introduced electrode with a "trocar shaped" extremity (thick arrow) to facilitate insertion and echogenic marks (white arrows) to improve US-guided tracking. (**c,d**) Custom-made plastic grids to accommodate electrodes. (**e**) During the procedure, these grids stabilise the array and prevent electrode displacement caused by muscle contraction; additionally, by maintaining electrode parallelism, the grids ensure the homogeneity of the electric field. In the presented case, a patient with metastatic Merkel cell carcinoma undergoes VEG-ECT for a large subcutaneous metastasis along with standard ECT for superficial metastases (*).

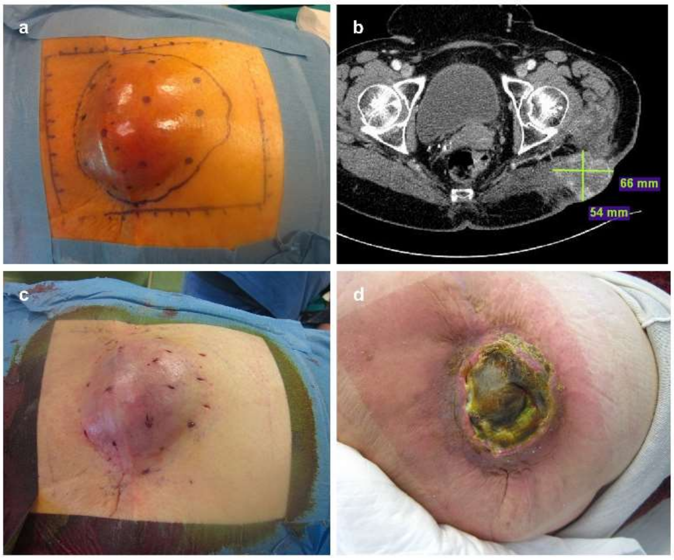


**Supplementary Figure S4.** Treatment toxicity. (**a**) Baseline clinical presentation of a soft tissue sarcoma patient with recurrent disease in the left buttock. (**b**) Preoperative TC scan. (**c**) Early toxicity. After the conclusion of the procedure, mild venous congestion was present, likely related to the immediate "vascular-lock" effect exerted by electric pulses. (**d**) Late toxicity. At one month, extensive tumour necrosis and skin ulceration were present that required antibiotic therapy, minor surgical debridement and prolonged wound dressing.

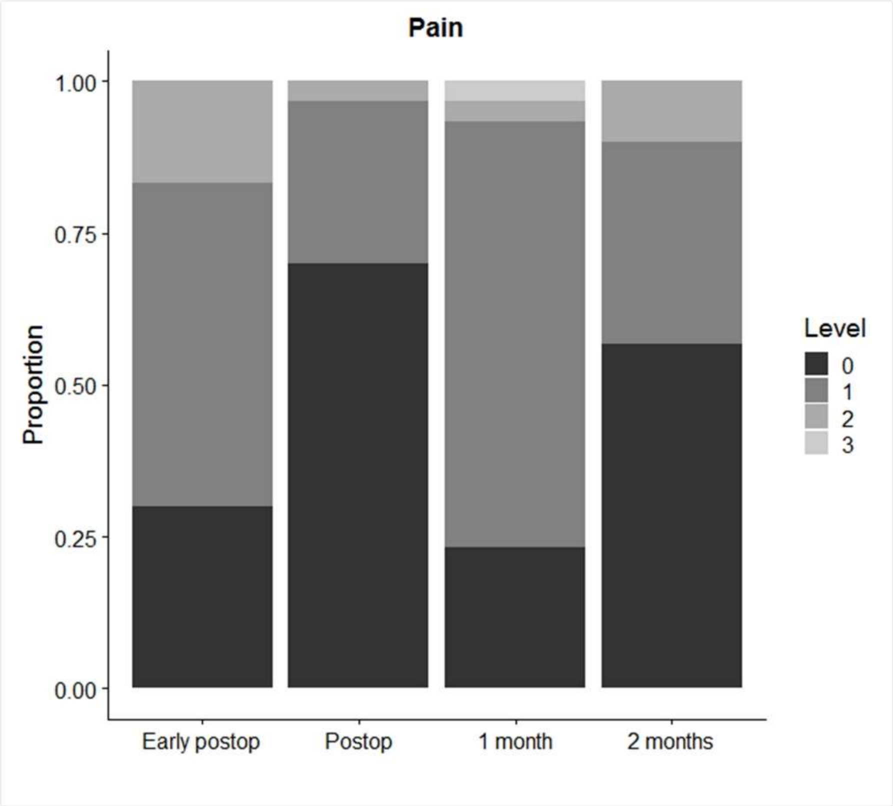


**Supplementary Figure S5.** Post-treatment pain. Mild or moderate pain was reported by 21/30 patients (70%) after VEG-ECT procedure, and by 9/30 patients (30%) during hospital stay (*P* = 0.02). At one month, 21/30 patients (70%) reported mild pain and 2/30 patients (6%) reported moderate or severe pain (*P* = 0.002). At two months, 13 patients (43%) had mild/moderate pain (*P* = 0.02). Legend: Early postop, pain evaluation performed in the recovery room at the end of the procedure; Postop, pain evaluation performed at 12/24 h, according to the length of hospital stay; Level, grading of pain according to the CTCAE v4.03 criteria (1 = mild; 2 = moderate; 3 = severe).

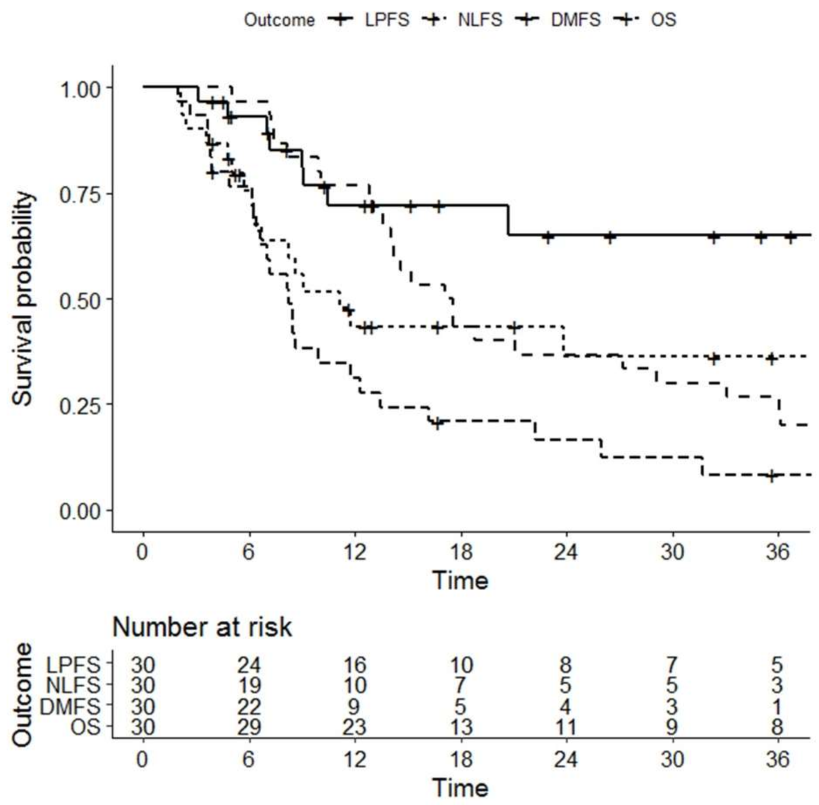


**Supplementary Figure S6.** Kaplan-Meier estimates of local progression-free survival (LPFS), new lesions-free survival (NLFS), distant metastasis-free survival (DMFS) and overall survival (OS). One- and 2-year survival rates were as follows: LPFS, 72% (95% CI 56%-92%) and 65% (95% CI 47%-89%), respectively; NLFS, 44% (95% CI 28%-68%) and 36% (95% CI 20%-64%); DMFS, 31%(95% CI 18%-54%) and 16% (95% CI 7%-38%); OS, 76%(95% CI 62%-93%) and 36% (95% CI 22%-58%).

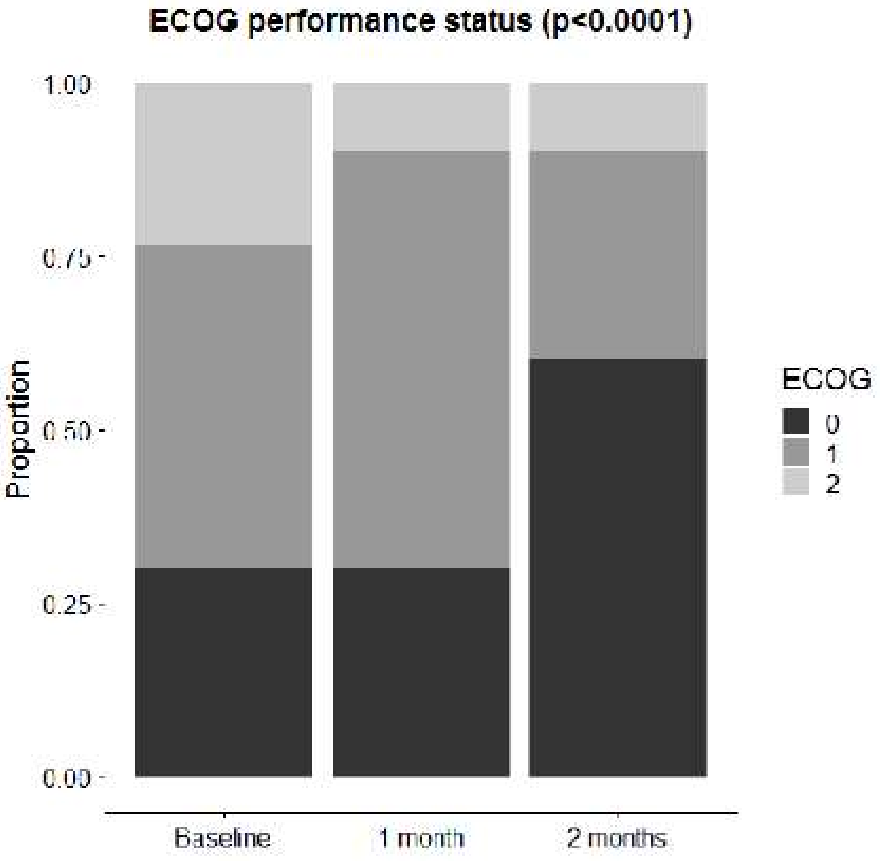


**Supplementary Figure S7.** Patient performance status, according to the Eastern Cooperative Oncology Group (ECOG) scale.

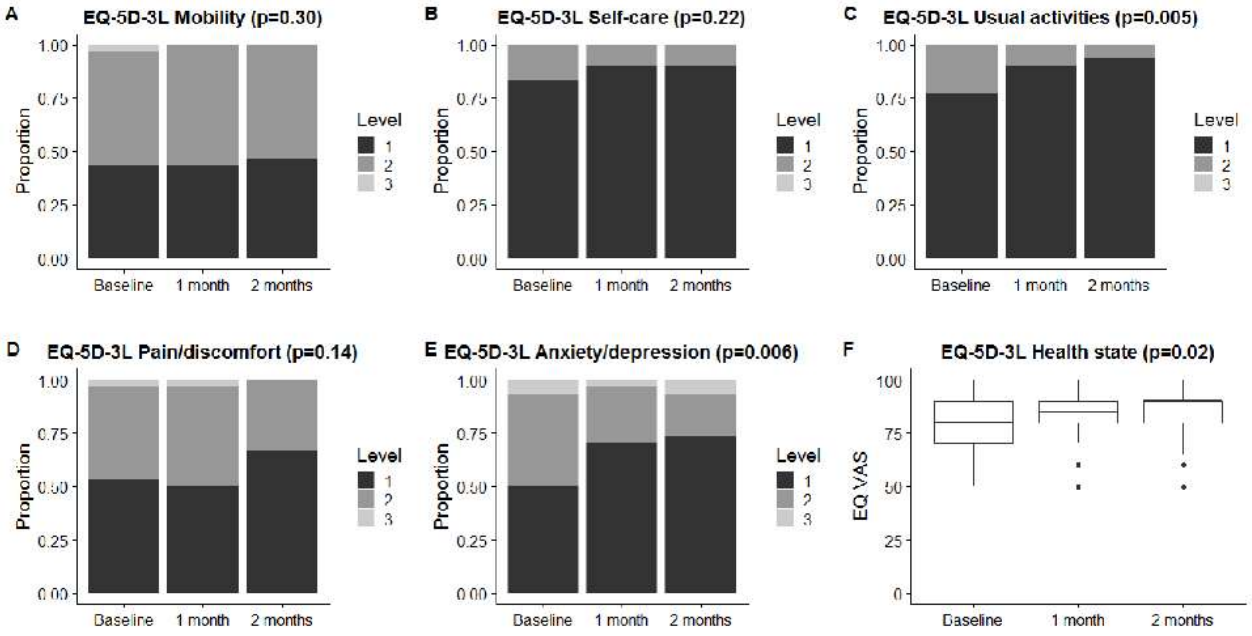


**Supplementary Figure S8.** Patient-reported health-related quality of life outcomes, according to the EuroQoL quality of life scale (EQ-5D-3L). The questionnaire includes five domains ("Motility", "Self-care", "Usual activities" "Pain/discomfort" and "Anxiety/depression"). Each domain was scored by patients on a 3-point Likert scale, where 1 = "I have no problems", 2 = "I have some problems", 3 = "I have extreme problems". Additionally, the "Overall health state" is scored on a visual analogue scale ranging from 0 (= worst imaginable health state) to 100 (= best imaginable health state).
